# Neuropeptide Y neurons surrounding the locus coeruleus inhibit noradrenergic system activity to reduce anxiety

**DOI:** 10.1126/sciadv.adq0011

**Published:** 2025-07-23

**Authors:** Danai Riga, Karlijn L. Kooij, Kelly Rademakers, Inge G. Wolterink-Donselaar, Onur Basak, Frank J. Meye

**Affiliations:** ^1^ Department of Translational Neuroscience, Brain Center, UMC Utrecht, Utrecht University, Utrecht, Netherlands.; ^2^ Department of Human Genetics, Amsterdam University Medical Center, Amsterdam, Netherlands.; ^3^ Department of Functional Genomics, Center for Neurogenomics and Cognitive Research, Vrije Universiteit Amsterdam, Amsterdam, Netherlands.

## Abstract

Adaptive responses to challenging environments depend on optimal function of the locus coeruleus (LC), the brain’s main source of noradrenaline and primary mediator of the initial stress response. Combining functional circuit dissection and causal in vivo interventions in mice, we here investigate a built-in peptidergic regulatory system that restricts LC noradrenergic output. In particular, we characterize a population of neuropeptide Y (NPY)–expressing neurons surrounding LC noradrenergic cells. We show that this peri-LC_NPY_ population exerts neuromodulatory inhibitory control over the LC via NPY-Y1R signaling. Under naïve conditions, this results in bidirectional control of anxiety-like behaviors. Stressful experiences recruit peri-LC_NPY_ neurons, leading to local NPY release in vivo, whereas enhanced peri-LC_NPY_ neuronal activity curbs anxiety after stress. Together, we establish a causal role for peri-LC_NPY_–mediated neuromodulation of the LC in the regulation of anxiety, providing mechanistic insights into the endogenous systems underlying adaptive responses to adversity.

## Teaser

Endogenous neuropeptide Y signaling modulates locus coeruleus activity, promoting adaptive responses to stress.

## INTRODUCTION

Adaptive responses to stressful experiences are paramount to our survival and well-being. Opposing systems that mediate initiation and termination of the stress response work in tandem to ensure optimal adaptation to challenging environments (*1*, *2*). The factors that dictate adaptive modulation of the stress response remain largely unknown, limiting available therapeutics against stress-related afflictions, such as anxiety disorders (*3*, *4*).

The locus coeruleus (LC) is the brain’s primary source of noradrenaline/norepinephrine (NE), and key regulator of the initial stress response (*5*–*7*). The LC_NE_ system mediates arousal and allocation of attention, preparing the organism for task-relevant and salience-specific responses, required in novel environments commonly associated with high cognitive and emotional load (*8*–*10*). To accomplish this, owing to its vast projection network and efferent collaterals (*11*), the LC coordinates myriad functions, from sympathetic responses, such as heart rate and pupil dilation, to complex, high-order cognitive processes, including goal orientation and decision-making (*9*). LC hyperactivity, characterized by high tonic firing, can lead to maladaptive responses to perceived threats, priming the development of pathological anxiety (*7*). In support, optogenetic stimulation of LC_NE_ neurons results in anxiety in mice, whereas chemogenetic inhibition of LC_NE_ cells strongly suppresses stress-driven anxiety-like behaviors (*12*). Given the importance of LC_NE_ activity in shaping anxiety responses, it is important to understand the mechanisms that appropriately regulate its function.

A strong candidate regulator of LC_NE_ activity is the neuropeptide Y (NPY) system, composed of groups of neurons that communicate via NPY release, and others that interpret these signals. NPY, one of the most widely distributed neuropeptides in the central nervous system, is traditionally associated with stress coping and anxiety relief (*13*–*15*), and it is dubbed the “stress resilience” molecule (*3*) after early preclinical studies, which used NPY or NPY receptor agonists, highlighted its anxiolytic effects (*16*–*19*). In support, variations in *NPY* gene expression and low NPY plasma levels have been linked to trait anxiety and stress-related neuropsychological conditions in clinical settings (*3*, *20*).

NPY immunoreactivity and NPY receptor presence have been observed in the LC (*21*, *22*). However, few studies have examined the functional relationship between (endogenous) NPY-mediated neuromodulation and the LC_NE_ system. Ex vivo electrophysiological evidence shows that exogenously applied NPY reduces LC_NE_ spontaneous discharge (*23*) and facilitates hyperpolarization of LC_NE_ neurons (*24*). These studies highlight an inhibiting effect of NPY on tonic LC firing, which could be crucial in regulating LC activity during arousal, and under stress. In agreement, exogenous NPY application aiming at the pericoerulean space, a region rich in noradrenergic dendritic fibers, induces anxiolysis in the elevated plus maze (EPM) (*25*), an innately anxiogenic behavioral task that is known to engage the LC (*26*).

Despite these insights, the endogenous source of NPY to the LC remains unidentified. In the rat brain, NPY-like immunoreactivity has been observed in LC_NE_ cell bodies as well as projection fibers (*21*, *27*–*30*), suggesting that NPY (co-)released by LC_NE_ neurons constitutes the main endogenous source of NPY to the LC. However, recent RNA sequencing data challenge this, demonstrating NPY presence in the pericoerulean space but no coexpression in noradrenergic neurons of the mouse LC (*31*, *32*). These contradictory reports have cast doubt on the origins of NPY input to the region. Furthermore, independently of its origins, no studies have addressed how endogenously released NPY modulates LC_NE_ activity to regulate anxiety levels. Understanding the effects of endogenous NPY signaling is critical as pharmacologically applied NPY engages distinct NPY receptors, resulting in dose-dependent, opposing regulation of anxiety-like behaviors (*33*).

Aiming to address this, we examined NPY organization and function in the mouse LC. We characterize a previously unidentified NPY-expressing, pericoerulean (peri-LC_NPY_) neuronal population at the anatomical, electrophysiological, and behavioral level. Our data indicate that peri-LC_NPY_ neurons constitute a distinct, non-noradrenergic population that directly suppresses LC_NE_ neuronal activity in an NPY-mediated, Y1 receptor–dependent manner. Moreover, we demonstrate that, under naïve conditions, activation of peri-LC_NPY_ cells reduces anxiety-like behaviors, via Y1 receptors, and conversely, peri-LC_NPY_ inhibition promotes anxiogenesis. Last, we show that exposure to stress engages peri-LC_NPY_ cells, increasing their excitability, and results in NPY release within the pericoerulean region. Notably, increasing local NPY availability by chemogenetic peri-LC_NPY_ neuron stimulation results in anxiety relief in animals previously exposed to stress. Together, we here describe a population of NPY-expressing cells that regulates the LC noradrenergic system, thereby promoting adaptive behavioral responses in arousing/adverse environments.

## RESULTS

### NPY-expressing neurons occupy the pericoerulean space

**Fig. 1.**
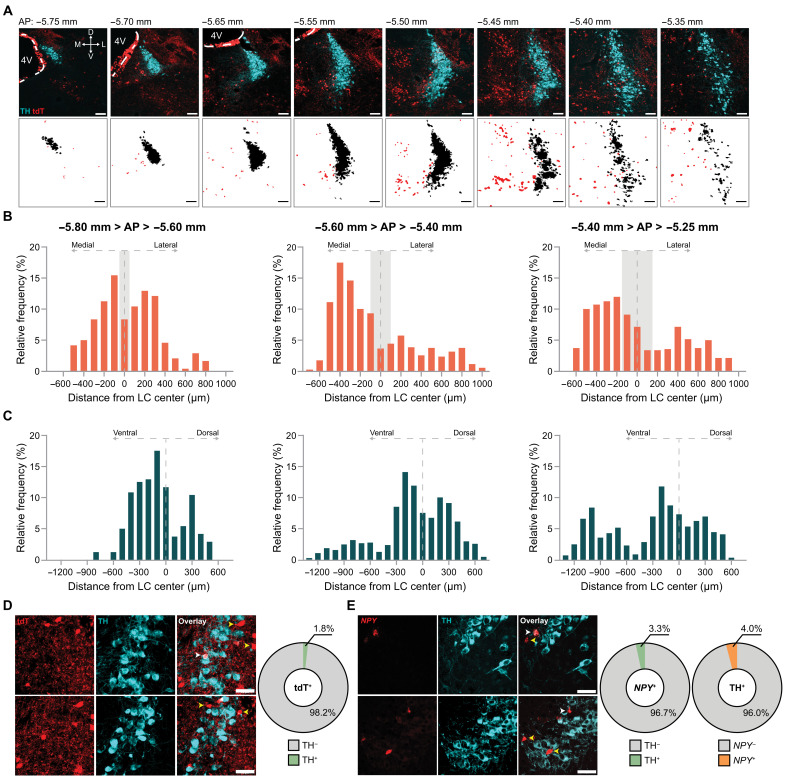
Peri-LC_NPY_ neurons are distributed medial to LC proper and are TH lacking. (**A**) Representative (top) and FIJI-processed (bottom) images of NPY^+^ (tdT, red) and NE^+^ (TH, cyan or black) cells in *NPY-cre:Ai14* mice. 4V, 4th ventricle. Scale bars, 100 μm. (**B** and **C**) Frequency of distribution (%) of peri-LC_NPY_ neurons location with respect to the LC center at the ML and DV axes. Gray shading, LC proper width. AP, −5.80 to −5.60 mm, *N* = 2, *n* = 239; AP, −5.60 to −5.40 mm, *N* = 7, *n* = 1004; AP, −5.40 to −5.25 mm, *N* = 6, *n* = 557. (**D**) Left: Representative examples of NPY^+^ (tdT, red) and NE^+^ (TH, cyan) cells in *NPY-cre:Ai14* mice. Right: Quantification of colocalization. TH expression in NPY^+^ neurons: TH^−^ cells (98.2%), yellow arrows; TH^+^ cells (1.8%), white arrows. Scale bars, 50 μm. *N* = 5, *n* = 612. (**E**) Left: Representative examples of *Npy^+^* (red), and NE^+^ (TH, cyan) cells in C57BL/6 mice. Right: Quantification of colocalization. TH expression in *Npy^+^* neurons: TH^−^ (96.7%), yellow arrows; TH^+^ (3.3%), white arrows. *Npy* puncta in TH^+^ neurons: *Npy*^−^ (96%), yellow arrows; *Npy*^+^ (4%), white arrows. Scale bars, 50 μm. *Npy*: *N* = 2, *n* = 30; TH: *N* = 3, *n* = 125.

To examine the presence of NPY neurons in the region, *NPY-cre* mice (*34*) were crossed with the *Ai14* reporter line (*35*), enabling tdTomato (tdT) fluorescence selectively in NPY-expressing cells (fig. S1A). We observed a clear presence of tdT^+^ cells neighboring the LC proper, the region occupied by noradrenergic cell bodies (*9*) (Fig. 1A and fig. S1B). We systematically surveyed peri-LC_NPY_ cell location with respect to noradrenergic neurons of the LC (LC_NE_), identified by tyrosine hydroxylase (TH) expression. In coronal slices immunolabeled for TH, we extracted the coordinates of tdT^+^ cells at the entire rostrocaudal axis [anterior-posterior (AP): −5.80 to −5.25 mm from bregma] containing the LC (Fig. 1A). We then used the distance from the LC center to plot peri-LC_NPY_ cell distribution at the mediolateral (ML) (Fig. 1B and fig. S2) and dorsoventral (DV) axes (Fig. 1C and fig. S2). Within the pericoerulean space, defined by the extent of reach of LC dendritic processes (fig. S1, B to E) (*36*), peri-LC_NPY_ neurons congregated largely medially (~60%) to the LC proper, with ~20% found dorsal and ~40% ventral to TH^+^ cell bodies.

Our topographical data indicated that, based on location alone, the peri-LC_NPY_ population is, to a large degree, distinct from LC_NE_ cells. To further validate this, in coronal slices from *NPY-cre:Ai14* mice immunolabeled for TH, we quantified the percentage of TH-expressing peri-LC_NPY_ cells in the total peri-LC_NPY_ population (Fig. 1D). In contrast to earlier studies, which showed large overlap between noradrenergic and NPY-expressing neurons (*21*, *27*), the vast majority of tdT^+^ cells were TH devoid, whereas only 1.8% coexpressed TH, indicating that the absolute number of NPY neurons occupying the pericoerulean space has been largely underestimated (*31*). To control for incomplete Cre-recombinase expression in *NPY-cre:Ai14* mice that could potentially confound these results, we used multiplex fluorescent RNAscope in situ hybridization (ISH) against endogenous *Npy*, in combination with TH immunolabeling, in brain slices from wild-type C57BL/6 mice (Fig. 1E). We quantified TH expression in *Npy*^+^ cells and vice versa, *Npy* puncta in LC_NE_ neurons. In agreement with our earlier results, only 3.3% of peri-LC_NPY_ neurons coexpressed TH, and similarly only 4% of LC_NE_ cells were *Npy*^+^.

The peri-LC region is rich in γ-aminobutyric acid (GABA)–expressing interneurons (*32*, *37*), and in cortical areas, NPY marks distinct classes of GABAergic cells (*38*, *39*). To further characterize peri-LC_NPY_ molecular make-up, we performed ViewRNA ISH against *Npy* and two genes (*Slc17a6* and *Gad2*) that encode common peri-LC glutamatergic (Vglut2) and GABAergic (Gad65) protein markers (*32*), in brain slices from wild-type C57BL/6 mice (fig. S3A). Quantification of (co)expression indicated that the largest percentage of *Npy*^+^ cells are exclusively peptidergic (*Slc17a6^−^*/*Gad2^−^*; 37.9%). In addition, we identified glutamatergic (*Slc17a6^+^*/*Gad2^−^*; 25.8%), GABAergic (*Slc17a6^−^*/*Gad2^+^*; 15.9%) and combinatorial (*Slc17a6^+^*/*Gad^+^*; 20.5%) *Npy*^+^ subpopulations.

Immunohistochemical colocalization studies against GABA in *NPY-cre:Ai14* mice further corroborated the data obtained by ISH. GABA-expressing peri-LC_NPY_ cells accounted for 27.4% of the total peri-LC_NPY_ population (fig. S3B). A subpopulation of GABA^+^ cells of the peri-LC expresses somatostatin (SST) (*32*); thus, we next examined whether peri-LC_NPY_ neurons are also positive for this GABAergic marker. In *NPY-cre:Ai14* mice, the vast majority (98.6%) of SST^+^ cells of the peri-LC colocalized with tdT^+^ neurons exclusively located at the ventromedial corner of the LC proper. On the other hand, only a small population (6.4%) of tdT^+^ cells was SST^+^, together indicating that peri-LC_NPY_ neurons only seldom coexpress this neuropeptide (fig. S2C). Last, medial to the LC proper, the laterodorsal tegmental area (LDTg) is a nucleus rich in cholinergic neurons (*40*), often taken along in peri-LC preparations (*32*). To exclude that peri-LC_NPY_ cells belong to the LDTg cholinergic system, we quantified cells expressing choline acetyltransferase (ChAT) in *NPY-cre:Ai14* mice. Colocalization studies showed that the vast majority (99%) of tdT^+^ neurons do not express ChAT, and conversely, a very limited portion (1.6%) of the ChAT^+^ population is tdT^+^ (fig. S2D). Together, our data verified the existence of an NPY-expressing neuronal population neighboring the LC. Peri-LC_NPY_ neurons are preferentially located medial to the LC core of LC_NE_ cell bodies, distributed within the LC dendritic zone. Furthermore, our findings suggest that the large majority of peri-LC_NPY_ neurons are not noradrenergic or cholinergic, whereas only a subset coexpresses glutamatergic and/or GABAergic markers.

### Peri-LC_NPY_ neurons innervate the pericoerulean space

Next, we aimed to reveal whether peri-LC_NPY_ neurons project within the region, to provide direct (NPY) input to LC_NE_ cells. To this end, we used Cre-dependent, virus-mediated antero- and retrograde labeling of peri-LC_NPY_ cells and mapped their neuroanatomical circuitry (*41*). The vast majority of NE dendritic processes are located outside the nuclear core (fig. S1C), where they receive extensive non-noradrenergic synaptic contacts (*36*). Thus, we first investigated whether peri-LC_NPY_ neurons project within the dendritic zone of the LC to permit signaling from NPY and its potential cotransmitters in the region.

**Fig. 2.**
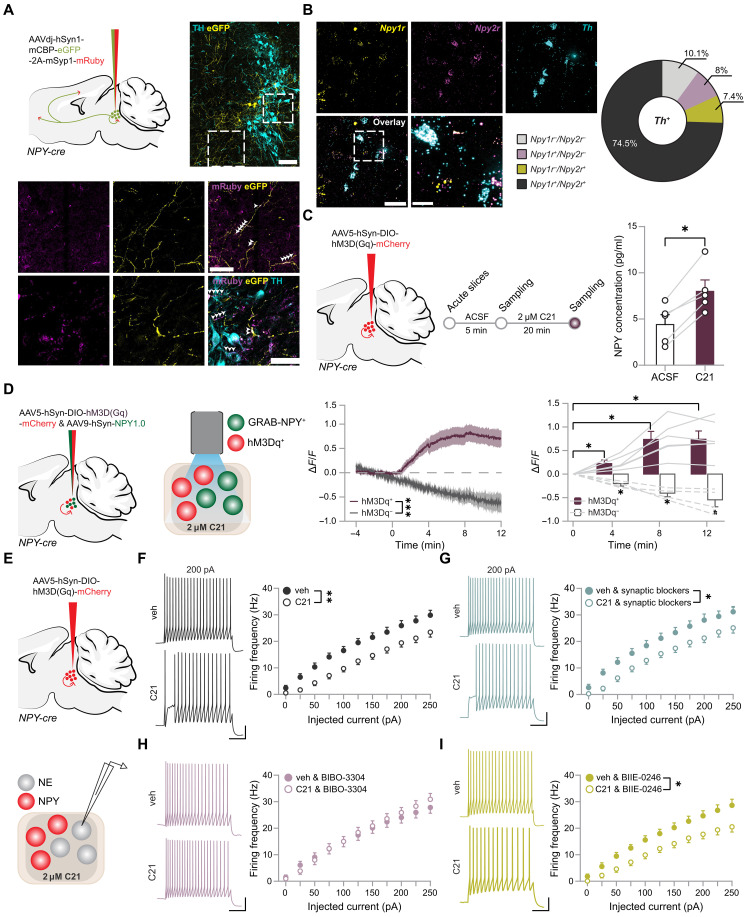
Peri-LC_NPY_ neurons reduce LC_NE_ excitability via Y1Rs. (**A**) Schematic of virus injections. Representative images of LC_NE_ immunolabeling (TH, cyan), GFP (yellow), and mRuby^+^ puncta (magenta, white arrowheads) along NPY^+^ axons. Scale bars, 100 and 50 μm (insets). *N* = 3. (**B**) Representative examples of *Npy1r* (yellow), *Npy2r* (magenta), and *Th* (cyan) mRNA in the LC of C57BL/6 mice. Quantification of *Npy1r* and *Npy2r* puncta in LC_NE_ neurons. Scale bars, 50 and 10 μm (inset). *N* = 4, *n* = 149. (**C**) Schematic of virus injections and experimental design for ELISA. Concentration of NPY before and after C21 (2 μM) treatment. Unpaired *t* test, *t*(8) = 2.44, *P* = 0.041. *N* = 5 replicates per treatment, *n* = 4 mice per replicate. (**D**) Schematic of virus injections. Average Δ*F*/*F* over time: two-way repeated measures (RM) ANOVA, time x group interaction *F*(3,24) = 27.73, *P* < 0.0001. Quantification of changes in GRAB_NPY_ Δ*F*/*F* from C21 superfusion (0 min) onward. Multiple comparisons (versus 0 min): hM3Dq^+^ 4 min, *P* = 0.024; 8 min, *P* = 0.013; 12 min, *P* = 0.015; hM3Dq^−^, 4 min, *P* = 0.019; 8 min, *P* = 0.017; 12 min, *P* = 0.064. hM3Dq^+^, *N* = 4 mice, *n* = 6 slices; hM3Dq^−^, *N* = 3 mice, *n* = 4 slices. (**E**) Schematic of virus injections. Recordings from LC_NE_ neurons in the presence of C21 or vehicle. (**F** to **I**) LC_NE_ firing frequency (Hz) per injected current (pA) and two-way RM ANOVA treatment effects: in the presence of C21, *F*(1,30) = 10.36, *P* = 0.003 (F); after pretreatment with synaptic blockers, *F*(1,25) = 6.18, *P* = 0.020 (G); after pretreatment with BIBO-3304, *F*(1,24) = 0.06, *P* = 0.815 (H); after pretreatment with BIIE-0246, *F*(1,21) = 7.41, *P* = 0.013 (I). Veh, *N* = 4, *n* = 15; C21, *N* = 4, *n* = 17. Veh + block, *N* = 2, *n* = 14; C21 + block, *N* = 3, *n* = 13. Veh + BIBO, *N* = 3, *n* = 13; C21 + BIBO, *N* = 4, *n* = 13. Veh + BIIE, *N* = 2, *n* = 11; C21 + BIIE, *N* = 3, *n* = 12. (F to I) Representative traces of LC_NE_ firing. Scale bars, 20 mV, 200 ms. Data are depicted as means ± SEM. ^#^*P* < 0.1; *P < 0.05; **P < 0.01; ***P < 0.001.

For this, we bilaterally injected *NPY-cre* mice with a Cre-dependent AVV (AAV-Syn-FLEX-CoChR-GFP) in the LC and allowed a period of ≥5 weeks of virus incubation. Next, we collected whole-brain slices and performed a qualitative analysis of labeled NPY^+^ cell bodies and fibers locally, within the LC, and in selected projection fields (fig. S4, A and B). Although we observed clear innervation of the pericoerulean space, we detected no peri-LC_NPY_ efferents in a selection of known LC output target regions (fig. S4, A and B). This suggests that local peri-LC_NPY_ neurons do not have long-range projection neuron characteristics, in contrast to NPY-expressing cells in certain other regions, such as the hippocampus (*42*). To further corroborate local connectivity of peri-LC_NPY_ neurons within the region, we next injected a Cre-dependent retrograde herpes simplex virus (HSV) (HSV-hEF1a-LS1L-mCherry) in the LC (fig. S4, C and D). This resulted in retrograde tracing of NPY^+^ cell bodies throughout the pericoerulean area, supporting the notion that peri-LC_NPY_ neurons terminate within the region. Moreover, we identified several brain areas containing LC-projecting NPY^+^ cells (fig. S4C and table S1). Both viral tracing strategies indicated that peri-LC_NPY_ projections assemble in the pericoerulean region. To assess whether these fibers are terminating or fibers of passage, we bilaterally injected an AAV construct (AAV-hSyn1-mCBP-EGFP-2A-mSyp1-mRuby) in the LC of *NPY-cre* mice for Cre-dependent expression of membrane-bound green fluorescent protein (GFP) and mRuby-fused synaptophysin, which enables axonal and presynaptic terminal labeling, respectively (*43*) (Fig. 2A). Using this approach, we identified mRuby^+^ puncta along peri-LC_NPY_ axons within both the LC nuclear core and dendritic zone (Fig. 2A), arguing for LC_NE_ synaptic innervation by local peri-LC_NPY_ cells. Together, these complementary tracing approaches indicate that peri-LC_NPY_ neurons can govern local neurotransmission.

### Peri-LC_NPY_ neurons do not form GABAergic or glutamatergic synaptic connections with LC_NE_ cells

On the basis of our anatomical data, we hypothesized that peri-LC_NPY_ neurons form functional synapses with LC_NE_ cells, necessary for LC neuromodulatory control. To test this, we bilaterally injected *NPY-cre* mice with a Cre-dependent AAV (AAV-Syn-FLEX-CoChR-GFP) in the LC, which enables selective expression of the highly conducting channelrhodopsin variant CoChR (*44*) in peri-LC_NPY_ neurons (fig. S5, A and B). After a period allowing for virus incubation and CoChR expression in terminals (≥5 weeks), we performed whole-cell patch-clamp recordings from LC_NE_ neurons, as identified based on their location and electrophysiological and morphological characteristics (*45*–*47*) (fig. S5C). In agreement with the prior literature (*45*), we confirmed that putative LC_NE_ neurons are inhibited by the α2 adrenergic receptor agonist clonidine (1 μM). Likewise, using biocytin-filling and post hoc immunolabeling against TH in a subset of recorded cells, we further validated cell NE identity (fig. S5C).

Next, to investigate the presence of direct peri-LC_NPY_→LC_NE_ synaptic connectivity, we photostimulated peri-LC_NPY_ neurons and recorded from LC_NE_ cells in brain slices with confirmed CoChR innervation (fig. S5, D and E). First, to detect α-amino-3-hydroxy-5-methyl-4-isoxazolepropionic acid receptor (AMPAR)–mediated or GABA A receptor (GABA_A_R)–mediated ionotropic currents, we optogenetically stimulated peri-LC_NPY_ neurons with single pulses while recording from LC_NE_ cells in voltage-clamp configuration at −50 mV. The fraction of LC_NE_ neurons that displayed perceptible postsynaptic responses to peri-LC_NPY_ photostimulation was negligible (AMPA-mediated, 0/39 cells; GABA_A_R-mediated, 1/39 cells). Rather, most LC_NE_ neurons remained unresponsive (38/39 cells; fig. S5D). In these experiments, we also applied trains of 20 pulses of 5-, 20-, or 50-Hz photostimulation to allow for the possibility of high-frequency stimulation requirements in detecting forms of fast-onset metabotropic signaling [e.g., GABA_B_R-mediated or mGluR (metabotropic glutamate receptor)–mediated] (*48*). As above, no such direct fast-onset responsivity was observed between peri-LC_NPY_ and LC_NE_ neurons (fig. S5D).

We performed the experiments above with a potassium gluconate (Kglu)–based intracellular solution, possibly underestimating connectivity at more distal inputs (*48*) to LC_NE_ neurons. To address this, we next recorded a subset of LC_NE_ cells using a cesium chloride (CsCl)–based internal in the absence of synaptic blockers, at −60 mV, to allow for the detection of GABA_A_R-mediated or eventual AMPAR-mediated currents (*48*). In agreement with our previous data, no responses to single optical pulse were observed (20/20 cells, nonresponsive; fig. S5E). Last, to account for the possibility of silent synapses, we performed recordings at +40 mV using the CsCl-based internal solution to detect *N*-methyl-d-aspartate receptor (NMDAR)–mediated currents. As before, no synaptic responses to peri-LC_NPY_ stimulation were seen (12/12 cells, nonresponsive; fig. S5E). Notably, the general absence of direct synaptic GABA/glutamate connectivity between the cell types was not due to an inability to pick up synaptic events. For instance, we observed a clear presence of spontaneously occurring inhibitory postsynaptic currents in the LC_NE_ cells that we recorded from (fig. S5F). Together, these experiments indicate that there is close to null glutamatergic or GABAergic ionotropic or fast-onset metabotropic synaptic input from peri-LC_NPY_ onto LC_NE_ cells.

### Pharmacologically applied NPY bidirectionally alters LC_NE_ neuronal excitability, via distinct NPY receptors

Communication between peri-LC_NPY_ and LC_NE_ neurons could be mediated by NPY itself and may exhibit a slower onset than detected in our synaptic connectivity study. To our knowledge, fast-onset synaptically driven, direct postsynaptic NPY receptor currents have not been observed before (*48*, *49*). Thus, we reasoned that peri-LC_NPY_→LC_NE_ cross-talk occurs in an alternative manner, other than direct time-locked synaptic currents. To further investigate this possibility, we first examined NPY receptor presence in LC_NE_ cells, which would be required to mediate direct NPY signaling. Using multiplex fluorescent RNAscope ISH, we identified LC_NE_ neurons that expressed *Npy1r* and/or *Npy2r* puncta in wild-type C57BL/6 mice (Fig. 2B). In accordance with a previous report (*30*), most LC_NE_ neurons expressed both receptors (*Npy1r*^+^/*Npy2r*^+^, 74.5%). Y1R (*Npy1r*^+^/*Npy2r*^−^, 8%)–expressing or Y2R (*Npy1r*^−^/*Npy2r*^+^, 7.4%)–expressing LC_NE_ subgroups were observed at a lesser extent, whereas the remaining fraction was YRs lacking (*Npy1r*^−^/ *Npy2r*^−^, 10.1%). Together, our data verified that LC_NE_ neurons express the necessary molecular machinery for NPY-mediated neuromodulation.

Next, we investigated whether changes in LC_NE_ intrinsic excitability could be a relevant readout for the effects of NPY. For this, we prepared brain slices from wild-type C57BL/6 mice, performed whole-cell patch-clamp recordings from LC_NE_ neurons, and bath-applied different doses of NPY or vehicle. In the presence of 30 nM NPY, we detected fewer action potentials in LC_NE_ neurons in response to increasing current injections, as compared to vehicle. This effect was mediated by Y1Rs as an NPY-induced decrease in LC_NE_ excitability was blocked in slices pretreated with the selective Y1R antagonist BIBO-3304 (*50*) (fig. S6). Unexpectedly, a 10-fold increase in NPY concentration yielded the opposite result as we observed LC_NE_ hyperexcitability in NPY-treated slices. Notably, LC_NE_ increased firing was abolished in the presence of the selective Y2R antagonist BIIE-0246 (*51*) (fig. S6). Furthermore, 300 nM NPY-induced LC_NE_ hyperexcitability was prevented in slices pretreated with synaptic blockers [for AMPA/kainate, NMDA, GABA_A_, and GABA_B_ receptors], suggesting that changes in LC_NE_ excitability may occur via an indirect network effect (fig. S6). Neither NPY dose affected the LC_NE_ neuron membrane resting potential (veh, −55.8 mV; 30 nM NPY, −61.7 mV; 300 nM NPY, −52.3 mV). Likewise, we observed no changes in membrane potential in the presence of YR antagonists or synaptic blockers (30 nM NPY + BIBO, −54.2 mV; 300 nM NPY + BIIE, −57.5 mV; veh + blockers, −51.9 mV; 300 nM NPY + blockers, −50.8 mV). Overall, these observations highlight the capacity of LC_NE_ neurons to respond, via different mechanisms, to pharmacologically applied NPY, with opposing effects on their excitability. This further emphasizes the need to decipher the substrates underlying endogenous NPY-mediated neuromodulation of the LC_NE_ system.

### Peri-LC_NPY_ neurons suppress LC_NE_ excitability via postsynaptic Y1Rs

Our pharmacological data demonstrated that NPY can bidirectionally control LC_NE_ excitability states, depending on the dose. However, it remained unclear whether endogenous peri-LC_NPY_–mediated signaling influences LC_NE_ firing properties, and if so, in which direction. To address this, we leveraged chemogenetics, which allow for protracted cell stimulation, to drive peri-LC_NPY_ activity and assessed the effects of local endogenous NPY signaling on the LC_NE_ system. For this, we bilaterally injected *NPY-cre* mice with a Cre-dependent AAV [AAV-hSyn-DIO-hM3D(Gq)-mCherry] to drive the expression of a Gq-coupled (excitatory) designer receptor exclusively activated by designer drugs (DREADD) in peri-LC_NPY_ cells. This viral construct enabled targeted chemogenetic activation of peri-LC_NPY_ neurons in the presence of the DREADD agonist compound 21 (C21) (*52*).

We established that chemogenetic stimulation engages the peri-LC_NPY_ population, resulting in local NPY release, using three ex vivo approaches (Fig. 2, C and D, and fig. S7, A and B). First, using patch-clamp electrophysiology, we showed that bath application of C21 (2 μM) increases peri-LC_NPY_ neuron firing in acute slices (fig. S7, A and B). Second, we used an enzyme-linked immunosorbent assay (ELISA) to biochemically detect NPY release in brain slices. We observed an increased concentration of NPY in the supernatant collected from C21-treated LC-containing slices (Fig. 2C). This effect was absent in slices treated with ACSF alone (fig. S7C). Last, we used a recently described GPCR-activation-based NPY (GRAB_NPY_) biosensor (*53*, *54*) to measure real-time NPY release upon peri-LC_NPY_ neuron stimulation. For this, we combined intra-LC viral delivery of the DREADD construct together with the GRAB_NPY_ biosensor (AAV9-hSyn-NPY1.0), which emits GFP signal upon NPY binding (*53*, *54*) (Fig. 2D). To validate that we can efficiently measure the GRAB_NPY_ signal in acute slice preparations, we first measured fluorescence changes upon bath application of NPY itself (300 μM; fig. S7D). NPY significantly increased GRAB_NPY_ Δ*F*/*F* response, which peaked at 8 min after NPY superfusion. Next, we assessed whether C21 would also increase the GRAB_NPY_ signal, reflecting endogenous NPY release in the region. In LC-containing brain slices that expressed both the DREADD and the GRAB_NPY_ constructs, C21 (2 μM) increased Δ*F*/*F* response that peaked at 8 min from the start of C21 superfusion (Fig. 2D). Validating the specificity of our observations, in slices void of hM3Dq-expressing peri-LC_NPY_ cells, we observed no fluorescence increase after C21, rather a reduction in Δ*F*/*F*, likely reflecting signal bleaching over time (Fig. 2D).

Having confirmed that chemogenetic stimulation of peri-LC_NPY_ neurons reliably triggers NPY release in slices, we then used ex vivo slice electrophysiology to examine its effects on LC_NE_ firing patterns. To this end, we proceeded with whole-cell patch-clamp recordings of LC_NE_ neurons in brain slices prepared from *NPY-cre* mice expressing the hM3Dq DREADD (Fig. 2E). Intrinsic excitability and passive electrophysiological properties of LC_NE_ cells were assessed in current-clamp configuration, after bath application (≥10 min) of C21 (2 μM) or vehicle (fig. S7E). In the presence of C21, we detected fewer action potentials in response to increasing current injections as compared to vehicle (Fig. 2F), mimicking the effect of low NPY dose application (cf., fig. S6). This effect, which occurred in a range of noradrenergic neuron firing frequencies, including those typically considered physiologically relevant (~10 Hz) (*55*, *56*), was accompanied by LC_NE_ membrane hyperpolarization (resting membrane potential: vehicle, −52.7 mV; C21, −63.5 mV) and an increased rheobase (vehicle, 15.0 pA; C21, 44.1 pA; fig. S7E), indicating that activation of local peri-LC_NPY_ neurons results in reduced LC_NE_ neuronal excitability. Peri-LC_NPY_ chemogenetic stimulation did not alter other electrophysiological parameters of LC_NE_ cells (fig. S7E). In brain slices that lacked hM3Dq expression in the region, we confirmed that C21 did not result in off-target, nonspecific effects on LC_NE_ neuronal firing (fig. S7F).

Next, we aimed to reveal the signaling mechanisms through which peri-LC_NPY_ neuron activity suppresses the excitability of LC_NE_ cells. In particular, we sought to address (i) whether peri-LC_NPY_ neurons exert direct or indirect [e.g., via GABAergic interneurons (*32*, *37*)] control over LC_NE_ neurons and (ii) whether this is mediated by NPY alone or by coreleased signaling molecules, such as GABA or glutamate (cf., fig. S3). To this end, we repeated the experiment of chemogenetic peri-LC_NPY_ neuron stimulation while measuring LC_NE_ neuron excitability, but this time we pharmacologically blocked synaptic network activity (i.e., blocking AMPAR/kainateR, NMDAR, GABA_A_R, and GABA_B_Rs; cf., fig. S6). Under these conditions, we still observed a clear decrease in LC_NE_ firing in the presence of C21 (Fig. 2G). These findings point toward direct postsynaptic effects of peri-LC_NPY_ neuron stimulation on LC_NE_ cell excitability, which are independent of GABAergic or glutamatergic intermediary cells.

Last, to examine whether C21 effects were specifically mediated by NPY signaling, we examined LC_NE_ excitability in the presence of selective Y1R or Y2R antagonists. In slices pretreated with the Y1R antagonist BIBO-3304 (1 μM), C21 effects on LC_NE_ excitability and membrane hyperpolarization were fully occluded (resting membrane potential: vehicle, −55.0 mV; C21, −60.6 mV; Fig. 2H and fig. S7E). On the contrary, blocking Y2Rs with BIIE-0246 (1 μM) did not prevent the ability of peri-LC_NPY_ stimulation to reduce LC_NE_ firing frequency (Fig. 2I). In the absence of C21, synaptic blockers, BIBO-3304, or BIIE-0246 alone did not alter LC_NE_ firing properties, further highlighting the specificity of the effects of peri-LC_NPY_ chemogenetic activation (fig. S7G).

Together, our data suggest that sustained stimulation of local peri-LC_NPY_ neurons releases NPY in the region, which hyperpolarizes LC_NE_ cells and dampens their excitability. Our observations point to postsynaptic Y1Rs, situated onto LC_NE_ neurons, being the prime mediators of local NPY signaling on the noradrenergic system. Complementing our synaptic connectivity studies (cf., fig. S5), these data indicate that NPY-driven neuromodulatory signals comprise the dominant form of communication between peri-LC_NPY_ and LC_NE_ neurons. Notably, chemogenetic activation of peri-LC_NPY_ neurons and exogenous application of 30 nM NPY (but not of 300 nM NPY) were analogous in regard to their effects on LC_NE_ intrinsic excitability, offering insights regarding the potential concentrations of chemogenetically evoked NPY release from peri-LC_NPY_ cells.

### Peri-LC_NPY_ neurons bidirectionally modulate anxiety-like behavior via local Y1Rs

**Fig. 3.**
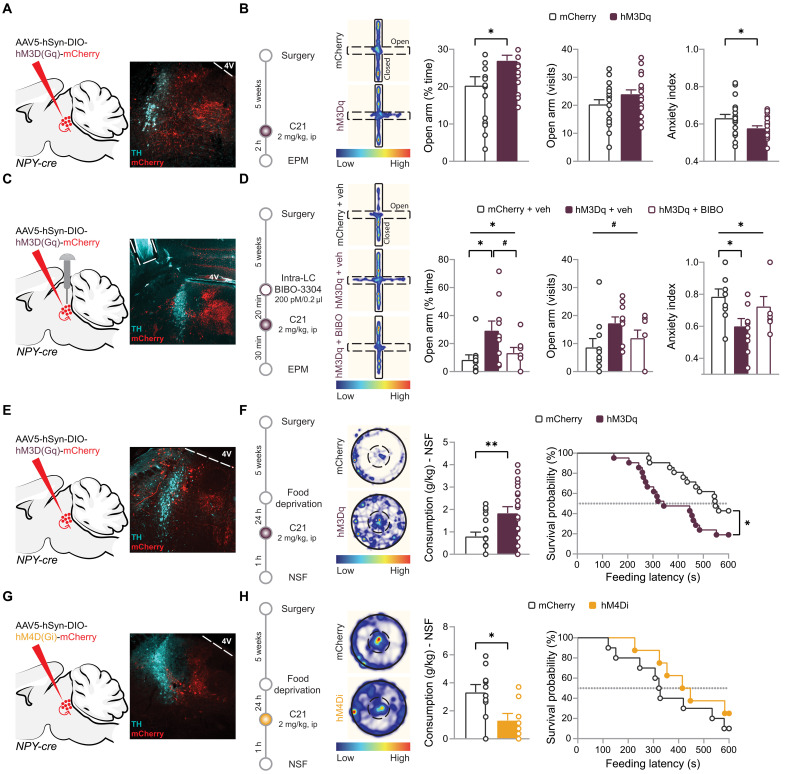
Peri-LC_NPY_ neurons bidirectionally regulate anxiety-like behavior. (**A**, **C**, **E**, and **G**) Schematic of injections for hM3Dq, hm4Di, or control virus and representative example of virus spread (mCherry, red) and cannula placement (dashed lines) in the LC (TH, cyan). (**B**) Experimental design and EPM performance after C21 administration. % time in open arms, mCherry, 20.3%; hM3Dq, 27%; unpaired *t* test, *t*(40) = 2.44, *P* = 0.019. Open arm entries, mCherry, 20.4; hM3Dq, 24; unpaired *t* test, *t*(40) = 1.67, *P* = 0.102. Anxiety index, mCherry, 0.63; hM3Dq, 0.58; unpaired *t* test, *t*(40) = 2.39, *P* = 0.021. mCherry, *N* = 21; hM3Dq, *N* = 21. h, hours. (**D**) Experimental design and EPM performance in vehicle and BIBO-3304 (200 pM/0.2 μl per side) pretreated mice, after C21 administration (2 mg/kg). % time in open arms, mCherry + veh, 8.5%; hM3Dq + veh, 29.4%; hM3Dq + BIBO, 13.4%; one-way ANOVA, *F*(2,25) = 4.86, *P* = 0.016; post hoc, hM3Dq + veh versus mCherry, *P* = 0.016; versus hM3Dq + BIBO, *P* = 0.097. Open arm entries, mCherry + veh, 8.7; hM3Dq + veh, 17.3; hM3Dq + BIBO, 12; one-way ANOVA, *F*(2,25) = 2.72, *P* = 0.085. Anxiety index, mCherry + veh, 0.79; hM3Dq + veh, 0.60; hM3Dq + BIBO, 0.73; one-way ANOVA, *F*(2,25) = 3.56, *P* = 0.044; post hoc, hM3Dq + veh versus mCherry, *P* = 0.037; versus hM3Dq + BIBO, *P* = 0.241. mCherry + veh, *N* = 10; hM3Dq + veh, *N* = 10; hM3Dq + BIBO-3304, *N* = 8. (**F** and **H**) Experimental design and NSF performance after C21 administration in hM3Dq (F) or hM4Di (H) groups. Food consumption: mCherry, 0.81 g/kg; hM3Dq, 1.84 g/kg; unpaired *t* test, *t*(40) = 3.03, *P* = 0.004; mCherry, 3.33 g/kg; hM4Di, 1.32 g/kg; unpaired *t* test, *t*(16) = 2.66, *P* = 0.017. Feeding latency: mCherry, 511 s; hM3Dq, 391 s; Mantel-Cox, χ^2^(1) = 5.52, *P* = 0.019; mCherry, 361 s; hM4Di, 444 s; Mantel-Cox, χ^2^(1) = 1.24, *P* = 0.265. mCherry, *N* = 21; hM3Dq *N* = 21 (F). mCherry, *N* = 10; hM4Di, *N* = 8 (H). (B, D, F, and H) Representative spatial location heatmaps. Data are depicted as means ± SEM. ^#^*P* < 0.1; *P < 0.05; **P < 0.01.

Optogenetic LC_NE_ neuron stimulation is anxiogenic at baseline conditions, and conversely, chemogenetic LC_NE_ neuron silencing prevents stress-induced anxiety (*12*, *57*, *58*). On the basis of the observed effects of peri-LC_NPY_ activation on LC_NE_ neuronal excitability, we next hypothesized that local NPY signaling could represent an endogenous mechanism responsible for the regulation of anxiety in novel and/or anxiogenic environments. To test this hypothesis, we examined the behavioral implications of peri-LC_NPY_ stimulation in vivo. We bilaterally injected *NPY-cre* mice with a Cre-dependent excitatory DREADD [AAV-hSyn-DIO-hM3D(Gq)-mCherry] or a vector expressing a control fluorescent protein (AAV-hSyn-DIO-mCherry) in the LC. After a period allowing for virus expression (5 weeks), mice were administered C21 [2 mg/kg, intraperitoneally (ip)] and were subjected to the EPM task to assess anxiety-like behavior (Fig. 3A). In hM3Dq mice, C21 increased the time spent in the open arms of the maze (mCherry, 20.3%; hM3Dq, 27%), indicating a reduction in baseline anxiety levels. This was accompanied by a decrease in anxiety index (mCherry, 0.63; hM3Dq, 0.58; Fig. 3B), a compound parameter that integrates avoidance and exploratory behaviors in the EPM (*59*). The reduction in anxiety-like behaviors occurred in the absence of altered general locomotion (distance moved: mCherry, 9.9 m; hM3Dq, 10.6 m; fig. S8A). Likewise, in a separate cohort of wild-type C57BL/6 mice, we confirmed that there were no off-target effects of systemic C21 on anxiety measures (fig. S8B).

Our findings suggest that NPY release by peri-LC_NPY_ neurons induces anxiolysis. We next assessed whether, in accordance with our electrophysiological data (cf., Fig. 2H), this effect was mediated by local Y1R activation. To address this, we performed an EPM experiment combining peri-LC_NPY_ chemogenetic activation with local, intra-LC Y1R antagonism in vivo. For this, *NPY-cre* mice expressing the excitatory DREADD [hM3D(Gq)] or mCherry control virus in peri-LC_NPY_ cells were bilaterally equipped with intracranial cannulas for LC-targeted administration of the Y1R antagonist BIBO-3304 (Fig. 3C and fig. S8C). After a period allowing for virus expression (≥5 weeks), we microinfused mice with BIBO (200 pmol/0.2 μl per side) or vehicle in the LC, before systemically administering C21 (2 mg/kg, ip) and then placed mice in the EPM (Fig. 3, C and D). As observed before, peri-LC_NPY_ stimulation was anxiolytic, whereas Y1 antagonism abolished this effect. In particular, vehicle-infused hM3Dq mice spent more time exploring the open arms of the EPM versus vehicle-infused mCherry controls and hM3Dq-expressing mice pretreated with the Y1R antagonist (mCherry + veh, 8.5%; hM3Dq + veh, 29.4%; hM3Dq + BIBO, 13.4%). Likewise, the hM3Dq + veh group displayed reduced anxiety index (mCherry + veh, 0.78; hM3Dq + veh, 0.60; hM3Dq + BIBO, 0.73) and a trend for increased open arm entries (mCherry + veh, 8.7; hM3Dq + veh, 17.3; hM3Dq + BIBO, 12; Fig. 3D). Unlike in the prior, noncannulated experiment (cf., Fig. 3, A and B, and fig. S8A), we here observed group effects on exploratory activity (distance moved: mCherry + veh, 5.6 m; hM3Dq + veh, 11 m; hM3Dq + BIBO, 7.2 m; fig. S8D). Nonetheless, our findings confirmed a causal link between peri-LC_NPY_ activity and the regulation of anxiety-like behaviors, where local Y1R-mediated NPY signal tempers LC_NE_ firing, promoting anxiolysis.

Our EPM data suggest that peri-LC_NPY_–mediated input to the LC drives anxiolysis. To further corroborate this, we evaluated the role of peri-LC_NPY_ neurons in another behavioral assay, namely, the novelty-suppressed feeding (NSF) test, which assesses hyponeophagia, the suppression of food intake due to exposure to a novel environment, as a proxy for anxiety (*60*). For this, we used the aforementioned cohort of *NPY-cre* mice with Cre-dependent excitatory DREADD (hM3Dq) or control virus (mCherry) in peri-LC_NPY_ neurons. Mice were food deprived for 24 hours, followed by systemic C21 administration (2 mg/kg, ip) and subjected to the NSF task (Fig. 3E). In hM3Dq mice, C21 decreased the latency to initiate consumption of a familiar food source (standard laboratory chow) placed at the center of an open field arena (mCherry, 511 s; hM3Dq, 391 s), indicating reduction in anxiety levels. In support, hM3Dq mice spent more time at the center of the arena (duration center: mCherry, 7.7%; hM3Dq, 13.4%), where they consumed significantly more food versus mCherry controls (NSF intake: mCherry, 0.81 g/kg; hM3Dq, 1.84 g/kg; Fig. 3F and fig. S8E). Controlling for effects of peri-LC_NPY_ chemogenetic stimulation on general consummatory behavior, we observed no group differences on food consumption in a familiar environment (home-cage intake: mCherry, 6.76 g/kg; hM3Dq, 7.35 g/kg; fig. S8E). Likewise, in a separate cohort of animals, we demonstrated that peri-LC_NPY_ stimulation does not affect home-cage chow intake in sated mice (fig. S8F). Last, no effects of C21 on general locomotor activity at the NSF were seen (distance moved: mCherry, 41.5 m; hM3Dq, 42.7 m; fig. S8E), validating the specificity of the anxiolytic effects of peri-LC_NPY_ activation.

Overall, our data support that peri-LC_NPY_ activation sufficiently drives anxiety relief. However, whether it is also required for anxiolysis remained unresolved. To address this, we next examined the effects of chemogenetic peri-LC_NPY_ inhibition on EPM and NSF tasks. For this, we made use of a Cre-dependent AAV [AAV-hSyn-DIO-hM4D(Gi)-mCherry] that drives the expression of an inhibitory DREADD. First, using electrophysiological recordings, we confirmed that hM4Di activation leads to peri-LC_NPY_ cells silencing (fig. S9, A and B). In particular, bath application of C21 (2 μM) resulted in reduced peri-LC_NPY_ spontaneous firing, recorded in current-clamp mode. Then, in an independent cohort of *NPY-cre* mice, we bilaterally injected the inhibitory DREADD or a control virus (AAV-hSyn-DIO-mCherry) in the LC. After a period allowing for virus expression (5 weeks), mice were food deprived (24 hours), administered C21 (2 mg/kg, ip), and then subjected to the NSF task, as described above (Fig. 3G). In hM4Di mice, C21 administration reduced food intake at the NSF arena, as compared to mCherry controls (NSF intake: mCherry, 3.33 g/kg; hM4Di, 1.32 g/kg), indicative of peri-LC_NPY_ inhibition-triggered anxiogenesis (Fig. 3H). This effect on food intake was specific for anxiogenic contexts as it did not occur when food was available at the home cage (home-cage intake: mCherry, 8.62 g/kg; hM4Di, 8.97 g/kg; fig. S9C). Peri-LC_NPY_ inhibition did not affect the latency to initiate food consumption in the NSF arena (mCherry, 361 s; hM4Di, 444 s) nor the time spent at its center (duration center: mCherry, 13.1%; hM4Di, 13.1%; Fig. 3H and fig. S9C). Likewise, C21 administration did not alter general locomotion in the hM4Di group (distance moved: mCherry, 40.8 m; hM4Di, 50.3 m; fig. S9C). Notably, peri-LC_NPY_ inhibition did not affect EPM performance (fig. S9D), indicating differential involvement of peri-LC_NPY_ inhibition in these two distinct anxiety tests. As we observed above for NSF, C21 did not affect locomotor activity in the EPM (distance moved: mCherry, 11.1 m; hM4Di, 11.5 m; fig. S9D). Together, our data support bidirectional control of (certain) anxiety-like behaviors by peri-LC_NPY_ neurons.

### Peri-LC_NPY_ neurons respond to acute stress, modulating stress-induced anxiety

The LC is an important regulator of the initial stress response, with implications for the development of stress-induced pathology (*7*). Given our data above, we predicted that peri-LC_NPY_ neurons would contribute to the neuromodulation of the LC_NE_ system not only under naïve conditions but also after stress to dampen its excitability, thereby curbing stress-induced anxiety. To test this hypothesis, we next examined whether stress engages peri-LC_NPY_ neurons and whether this leads to NPY release in the region.

First, we established a stress protocol [exposure to repeated electrical foot shocks (FS)] that drives LC activation (fig. S10, A to C) and results in subsequent anxiety-like phenotypes (fig. S10, D to F). In mice subjected to FS, we observed increased LC_NE_ cell excitability, shown in a higher number of action potentials in response to increasing current injections, as compared to nonstressed controls (NS; fig. S10B). This was accompanied by a reduction in the current necessary for LC_NE_ cells to exceed their action potential threshold and fire (rheobase: NS, 25 pA; FS, 0 pA; fig. S10C and table S2), further corroborating LC_NE_ engagement after stress. Stress-induced LC_NE_ system activation was in parallel with increased anxiety-like behaviors as FS mice showed decreased time spent in the open arm of the EPM compared to controls (NS, 23.0% versus FS, 13.8%), as well as an increase in anxiety index (NS, 0.62 versus FS, 0.68; fig. S10E). Notably, stress-induced anxiety was long-lasting, as reflected in reduced exploration of an open field 1 week following exposure to FS (fig. S10F). In particular, FS mice spent less time at the center of the open field arena (NS, 4%; FS, 2.5%), which they visited less frequently compared to controls (NS, 24.5 versus FS, 17.5).

**Fig. 4.**
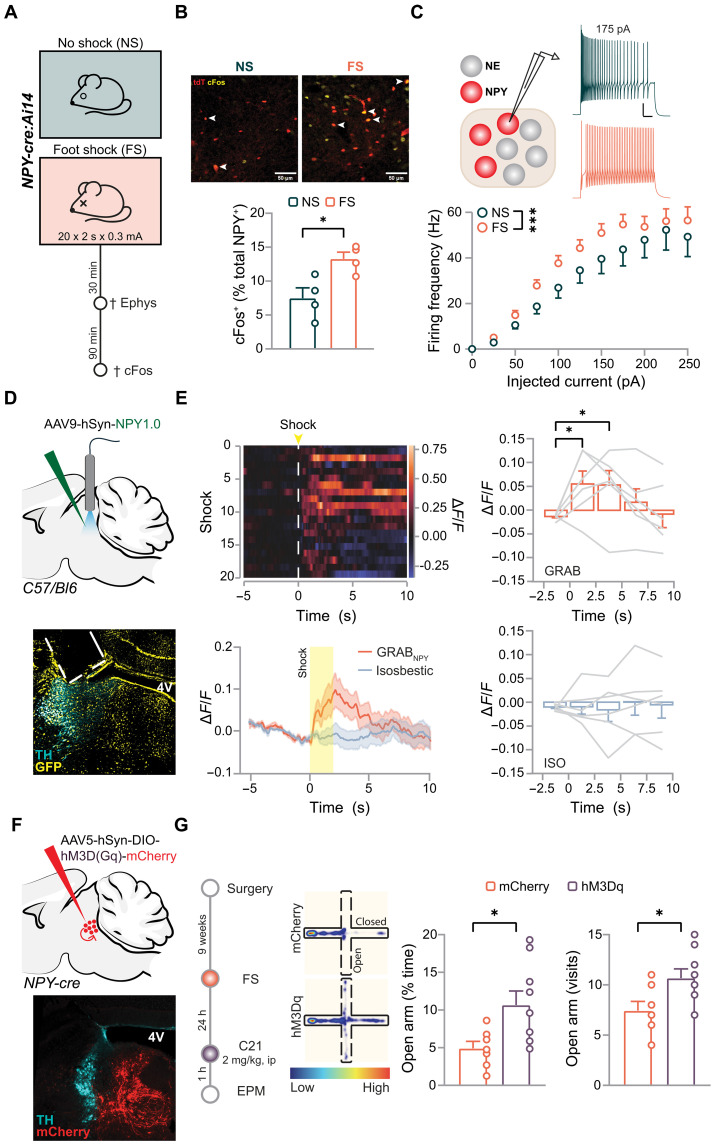
Stress recruits peri-LC_NPY_ neurons, resulting in local NPY release, and peri-LC_NPY_ neuron stimulation relieves anxiety after stress. (**A**) Experimental design for stress experiments. (**B**) Representative examples of tdTomato (tdT, red) and cFos immunolabeling (yellow) in NS and FS mice. Double-immunoreactive cells are indicated (white arrows). Scale bars, 50 μm. Quantification of cFos^+^ neurons (% of tdT^+^ cells); unpaired *t* test, *t*(6) = 3.169, *P* = 0.019. NS, *N* = 4, *n* = 1572; FS, *N* = 4, *n* = 867. (**C**) Recordings from peri-LC_NPY_ neurons in NS and FS groups. Representative traces of peri-LC_NPY_ firing. Scale bar, 20 mV, 100 ms. Peri-LC_NPY_ firing frequency (Hz) per injected current (pA). Two-way RM ANOVA main group effect, *F*(1,900) = 11.46, *P* = 0.001. NS, *N* = 10, *n* = 35; FS, *N* = 13, *n* = 46. (**D**) Schematic of injections and representative example of virus spread (GFP, yellow) and placement of fiber optics (dashed lines) in the LC (TH, cyan). (**E**) Representative heatmap of Δ*F*/*F* response time-locked to FS delivery. Average Δ*F*/*F* over time. GRAB_NPY_, RM ANOVA, time effect *F*(4,20) = 3.88, *P* = 0.017; Isosbestic, RM ANOVA, time effect *F*(4,20) = 0.214, *P* = 0.927. Quantification of changes in Δ*F*/*F* from shock onset (0 s) for GRAB_NPY_ and isosbestic channels. GRAB_NPY_ 0 to 2.5 s, paired *t* test, *t*(5) = −2.65, *P* = 0.046; 2.5 to 5 s, Wilcoxon signed-rank test, *W*(6) = 20, *P* = 0.046. *N* = 6. (**F**) Schematic of injections and representative example of virus spread (mCherry, red) in the LC (TH, cyan). (**G**) Experimental design, representative spatial location heatmap, and EPM performance in stress-exposed hM3Dq and control groups. Time in open arms (%), mCherry, 4.9%; hM3Dq, 10.7%; unpaired *t* test, *t*(14) = 2.53, *P* = 0.024. Open arm entries, mCherry, 7.4; hM3Dq, 10.7; unpaired *t* test, *t*(14) = 2.43, *P* = 0.029. mCherry, *N* = 7; hM3Dq, *N* = 9. Data are depicted as means ± SEM. *P < 0.05, ***P < 0.001.

Next, we examined whether our stress protocol recruits peri-LC_NPY_ cells (Fig. 4A). We quantified cFos expression, as proxy for neuronal activation, in peri-LC_NPY_ cells from control and stress-exposed mice (Fig. 4B). We found an increased number of cFos^+^ peri-LC_NPY_ cells in mice subjected to FS (NS, 7.5%; FS, 13.3%), corroborating the involvement of peri-LC_NPY_ neurons in the initial stress response. To further validate these results, we recorded peri-LC_NPY_ intrinsic properties in LC-containing slices prepared from *NPY x Ai14* control and FS-exposed mice (Fig. 4C). Stress did not affect peri-LC_NPY_ passive properties, such as cell capacitance and membrane resistance (table S2). Likewise, action potential threshold and resting membrane potential remained unaltered after exposure to stress (table S2). Control peri-LC_NPY_ neurons showed sustained capacity for high-frequency firing rates, corresponding well to the physiological requirements for neuropeptidergic release from dense core vesicles (*61*, *62*). Notably, in stressed mice, we detected an even greater number of action potentials in response to increasing current injections, as compared to controls (Fig. 4C), indicating peri-LC_NPY_ neuron engagement in stressful environments.

Our patch-clamp data suggested that stress triggers peri-LC_NPY_ activation ex vivo; however, whether this translates to increased NPY signaling in the region remained unknown. To address this, we next recorded real-time FS-induced NPY release in freely behaving mice using fiber photometric GRAB_NPY_ recordings. For this, we unilaterally injected the GRAB_NPY_ sensor in the LC of C57BL/6 mice subjected to FS stress and recorded NPY release upon shock delivery (Fig. 4D). FS resulted in increased Δ*F*/*F* response of GRAB_NPY_ from the shock onset and up to 5 s postshock (Fig. 4E). Conversely, FS exposure did not alter Δ*F*/*F* response in mice expressing a GFP (AAV-hSyn-eGFP) control construct (fig. S10G). Together, these data indicate that aversive stimuli drive NPY signaling within the pericoerulean space.

Together, our ex vivo and in vivo findings are in agreement with peri-LC_NPY_ neurons being recruited by stress, resulting in acute NPY availability in the region. We next investigated whether this NPY-mediated LC-targeted neuromodulatory signal plays a role in stress-evoked anxiety. For this, we exposed *NPY-cre* mice expressing the excitatory DREADD (hM3Dq) or a control vector (mCherry) in peri-LC_NPY_ neurons to FS (Fig. 4F) to induce stress-associated increases in LC_NE_ excitability and anxiety-like phenotypes (cf., fig. S10, A to F). Twenty-four hours following stress, mice were administered C21 (2 mg/kg, ip) and subjected to an EPM test. In the hM3Dq group, C21 increased the time spent in the open arms of the arena (mCherry, 4.9% versus hM3Dq, 10.7%) as well as open arms entries (mCherry, 7.4 versus hM3Dq, 10.7; Fig. 4G). This was accompanied by increased explorative behavior in the EPM (distance moved: mCherry, 5.1 m versus hM3Dq, 7.5 m; fig. S10H). In addition, we observed a trend for reduced anxiety index in hM3Dq mice (mCherry, 0.72 versus hM3Dq, 0.66; fig. S10H). Together, these data indicate that, similar to naïve conditions, LC NPY signaling induces anxiolysis after stress.

## DISCUSSION

Despite decades of research on the basic mechanisms underlying adaptive stress reactivity, our knowledge of the neural circuitries and molecular substrates that counteract stress effects remains incomplete. The LC_NE_ system is the brain’s “first responder” to stress and primary coordinator of the global neural processes underlying flight-or-fight (*7*, *9*, *63*). We reasoned that elucidating the built-in systems that restrict LC function could provide us with a mechanistic understanding of how to promote adaptive responses to challenging environments. For this, we examined the endogenous mechanisms underlying NPY-mediated LC neuromodulation. Collectively, we here demonstrate that a previously uncharacterized population of NPY-expressing cells, situated in the pericoerulean region, provides direct Y1R-mediated inhibitory control over LC noradrenergic neurons. Under approach-avoidance conflicts, peri-LC_NPY_–mediated neuromodulation of the LC efficiently lowers anxiety levels, via Y1R-dependent mechanisms. Conversely, suppressing local peri-LC_NPY_ neuronal activity results in anxiogenesis. Last, we show that acute stress engages peri-LC_NPY_ neurons and results in in vivo NPY release in the region. In turn, enhancing peri-LC_NPY_ cell activity relieves stress-induced anxiety. Together, our findings highlight a role for peri-LC_NPY_ neurons as key mediators of LC function and delineate an endogenous circuit for the regulation of anxiety.

### An NPY-expressing population envelops the LC

We report that the large majority of peri-LC_NPY_ neurons are situated medially to the LC nuclear core, within a region that is rich both in noradrenergic dendritic fibers and diverse axon terminals targeting them. Convergent signals within this area, mediated by fast neurotransmission or slow peptidergic input, are proposed to coordinate (modular) LC engagement in arousal and under stress (*12*, *37*, *64*–*66*). Thus, peri-LC_NPY_ neurons are ideally situated to fine-tune LC_NE_ activity levels when required. Our neuroanatomical tracing data support this notion as we provide evidence that peri-LC_NPY_ neurons project to the LC proper and its dendritic zone, forming functional contacts within the region and with LC_NE_ neurons themselves. Viral anterograde tracing did not reveal peri-LC_NPY_ neuronal efferents in other primary LC output regions. However, given that peri-LC_NPY_ cells comprise a relatively small population, we cannot exclude that unbundled projection fibers remained undetected in our preparations. Furthermore, our retrograde tracing data identified several brain regions that project to the LC, indicating that peri-LC_NPY_ cells are not the sole source of NPY in the region. Future studies are required to independently validate the specificity of these inputs and unravel non-LC NPY contributions to the neuromodulation of the LC noradrenergic system.

In contrast to earlier studies in rats (*21*, *28*, *67*), we here report that peri-LC_NPY_ neurons constitute a distinct, predominantly non-noradrenergic neuronal population. Although species differences might contribute to the observed discrepancy, it is also plausible that methodological limitations previously overestimated NPY-NE colocalization in the LC proper. Recent single-cell and single-nucleus RNA sequencing data corroborated the lack of NPY-expressing noradrenergic neurons, both in the mouse (*31*, *68*) and in the human (*69*) LC. Notably, similar to our observations, NPY transcripts were detected outside the LC nuclear core (*31*, *32*), further validating the existence of a previously undetected discrete pool of NPY-expressing neurons populating the mouse pericoerulean space. Peri-LC_NPY_ cells only partially coexpress fast neurotransmitters, further dissociating them from the diverse GABAergic populations occupying the peri-LC (*70*). Local GABA input to the LC has been implicated in physiological functions essential to the stress response, such as arousal (*37*). Unlike peri-LC_NPY_ neurons, stimulation of peri-LC GABA cells does not directly drive anxiolysis; however, inhibition of these neurons increases anxiety-like behaviors (*32*), likely by lifting a local inhibitory brake on LC_NE_ cells. It is thus possible that, under adversity, NPY-mediated neuromodulation acts synergistically with fast GABAergic signaling to exert complementary control over the LC. Of note, we cannot exclude that peri-LC_NPY_ neurons target also other neurons than LC_NE_ cells within the pericoerulean space. Future studies are required to delineate such microcircuitry and its functional relevance in stress and anxiety.

### Peri-LC_NPY_ neurons directly inhibit LC_NE_ activity via NPY-Y1 receptor signaling

Our combined ex vivo chemogenetic, biochemical, biosensor, and electrophysiological data strongly support the notion that peri-LC_NPY_ neurons comprise a source of selective NPY-mediated neuromodulatory input to the LC that reduces LC_NE_ excitability. Although our data indicate that, aside from NPY transmission, there is little to no fast GABAergic and glutamatergic transmission between peri-LC_NPY_ and LC_NE_ neurons, we cannot exclude potential co-occurrence of alternative forms of neurotransmission (e.g., glycine-mediated transmission) between these cell types. However, we show that the ability of peri-LC_NPY_ neurons to suppress LC_NE_ neurons requires NPY Y1Rs, which, according to our ISH data, are localized postsynaptically on LC_NE_ neurons. Y1Rs’ precise subcellular localization on LC_NE_ neurons that receive NPY input remains to be determined and could include dendritic sites (e.g., at the LC dendritic zone), cell bodies (e.g., at the LC proper), or both (*30*, *71*).

Moreover, the exact molecular mechanisms through which NPY alters LC_NE_ excitability remain to be addressed. We detect LC excitability tempering effects that resemble that of exogenous NPY application in the lateral amygdala (*72*). Amygdalar projection neurons respond to pharmacologically applied NPY with hyperpolarization, an effect mediated by Y1 (and not Y2) receptors, via activation of G-coupled inward rectifying potassium (GIRK) channels (*72*). Altered GIRK conductances could be a potential effector in the case of NPY regulation of LC_NE_ neurons. Notably, following peri-LC_NPY_ chemogenetic activation, we did not detect changes in LC_NE_ membrane resistance, calculated near the resting membrane potential. However, we cannot exclude differences in potassium channel–regulated membrane resistance at more positive potentials, which we did not assess here as, in our experiment, those coincided with action potential–driven active conductances.

In accordance with the Y1R-dependent control of peri-LC_NPY_ neurons over LC_NE_ excitability, we here demonstrate that the anxiety-relieving effects of local NPY release in the LC are mediated via Y1Rs. Our combined ex vivo (acute slices) and in vivo (EPM) observations are in line with a mechanism whereby peri-LC_NPY_–mediated local NPY neuromodulatory tone inhibits the LC to promote anxiolysis via Y1Rs. Our data are in conflict with an earlier study in rats (*25*), where pharmacologically applied NPY within the LC vicinity resulted in reduced anxiety via Y2Rs. In particular, Kask and colleagues showed that infusion of NPY or an agonist with affinity for Y2/Y5 receptors in the pericoerulean space had an anxiolytic effect in rats performing the EPM task (*25*). On the contrary, a singular low dose of a Y1R/Y5R agonist did not produce this effect. These findings seem at odds with our observations that implicate Y1Rs in the regulation of anxiety-like behaviors following chemogenetically evoked release by local NPY sources. Besides the species differences, there are several other discrepancies between the two studies.

First, exogenously applied NPY can induce widespread recruitment of Y2R-containing efferents outside the LC dendritic zone, with extra-LC contributions accounting for the observed behavioral effects. Instead, we here assessed the contribution of local pericoerulean NPY sources, which selectively and directly affect postsynaptic LC_NE_ intrinsic excitability, resulting in the ensuing Y1R-dependent anxiolysis. Second, pharmacological dose chosen could contribute to the apparent disparity between the two studies. We report that the pharmacological effects of exogenously applied NPY on LC_NE_ activity are dose dependent. Bath-applied NPY modulates LC_NE_ neurons in opposite directions (hypo- versus hyperexcitability), via distinct NPY receptors. In particular, low NPY dose mimicked the effects of chemogenetic activation of peri-LC_NPY_ neurons, with both depending on postsynaptic Y1Rs. High NPY dose led to opposite effects onto LC_NE_ cells, involving Y2Rs and a network effect. Although we did not specifically test this, it is possible that presynaptic Y2Rs, expressed on peri-LC_NPY_ cells, play an autoregulatory role (*73*), in which they limit peri-LC_NPY_ signaling, leading to disinhibition of LC_NE_ neurons and increased LC_NE_ firing frequency. During adversity, increased Y2R-mediated LC reactivity might drive flight-or-fight responses, and this is in agreement with the well-described role of Y2Rs in anxiogenesis (*74*–*77*). On the other hand, in arousing, but not threatening, environments, limiting the initial stress response by Y1R-mediated LC silencing might preclude the development of maladaptive behavioral patterns. In several brain regions other than the LC, Y1R activation results in anxiolysis (*17*, *19*, *78*–*80*). Collectively, these results highlight the intricate outcomes of NPY-mediated neuromodulation of the LC_NE_ system, where NPY tone can fine-tune LC_NE_ activity toward both hypo- and hyperexcitation. Which physiological conditions prompt each requirement in vivo remains to be determined.

### Functional role of peri-LC_NPY_ neurons in anxiety

Combining in vivo chemogenetic manipulations with behavioral assessment, we here showed that modulation of peri-LC_NPY_ neuronal activity exerts bidirectional control over anxiety-like phenotypes under naïve conditions. In particular, in vivo peri-LC_NPY_ stimulation, which acutely increases NPY availability in the region, increased exploration/approach in the EPM and NSF tasks. This is in agreement with peri-LC_NPY_–mediated silencing of LC_NE_ cells and, presumably, reduced NE release in LC output regions to promote anxiolysis. Likewise, we demonstrate that peri-LC_NPY_ inhibition, which is expected to lift the NPY-mediated brake on LC_NE_ neurons, led to anxiogenesis in the NSF task. We report a lack of effect of in vivo peri-LC_NPY_ inhibition in the EPM, implying divergent requirements for peri-LC_NPY_ function in a task-specific manner. Of note, in the NSF, but not the EPM, mice were subjected to prior food deprivation, which is shown to increase LC neuronal activity during food approach (*58*). Increased LC engagement could set the stage for enhanced peri-LC_NPY_ recruitment upon fasting. Thus, our NSF data might reflect that upon food scarcity, (basal) peri-LC_NPY_ activity partially offsets increased LC_NE_ engagement and its associated avoidance/aversion (*12*, *58*), thereby promoting exploration and foraging in new, and potentially unsafe, environments. In accordance with this notion, whereas peri-LC_NPY_ activity levels influenced food drive and consumption in the anxiogenic NSF task, it did not affect consummatory behavior in the familiar (safe) context of the home cage or under conditions of satiety.

In addition to addressing the implication of peri-LC_NPY_ neurons in the regulation of basal anxiety, we investigated their role under adverse conditions, which engage the LC_NE_ system leading to lasting anxiety-like phenotypes. The role of NPY signaling in stress relief is well documented (*13*), but only sparse literature has linked this to the LC. For example, physical stress (restraint, followed by forced swimming) elevates *Npy*, *Npyr1*, and *Npyr2* mRNA expression in the region (*81*, *82*), indicating recruitment of the local NPY system during adversity. These data are in accordance with our findings of increased firing frequency and increased cFos in peri-LC_NPY_ neurons after FS stress. Our in vivo fiber photometric experiments with the GRAB_NPY_ sensor further corroborate that NPY output to the LC is acutely increased after stress, in awake, behaving animals. One limitation of the use of this biosensor is that, thus far, its sensitivity and specificity have mainly been validated in vitro (*53*, *54*). Further studies definitively showing that these aspects also apply when the sensor is used in vivo (e.g., using local pharmacological receptor blockade or knockout) remain important. Notably, in the current study, the biosensor data conclusions are supported by several other independent experimental approaches. Last, although we cannot exclude contribution of non-LC NPY inputs to the observed effects, our combined ex vivo and in vivo data argue for the local peri-LC_NPY_ population being a prominent source of NPY-mediated neuromodulation of the LC under stress. In agreement, we showed that chemogenetic manipulation of peri-LC_NPY_ cells reduces anxiety in animals previously subjected to stress, possibly by alleviating stress-induced changes in LC_NE_ system responsivity.

In the current study, we performed cell type–specific dissection of an LC circuitry mediating anxiety relief. In particular, we provide mechanistic data for the anxiolytic effects of a previously unidentified NPY-expressing neuronal population. Using complementary approaches at different levels of analysis (i.e., cellular, circuit, and intact animal), we provide anatomical, electrophysiological, and behavioral evidence that peri-LC_NPY_ neurons exert modulatory control over the LC_NE_ system. Our combined results causally link endogenous NPY-mediated neuromodulation to adaptive LC engagement during arousal or upon adversity. In addition, we provide evidence for real-time, stress-driven NPY dynamics within the LC, linking local NPY signaling to the regulation of the initial stress response. Collectively, these data expand our understanding of how endogenous peptidergic influences regulate stress systems in the brain and highlight peri-LC_NPY_ neurons as a possible target for the modulation of (stress-induced) anxiety-like behaviors.

## METHODS

### Animals

In all experiments, naïve adult male or female mice were used (20 to 35 g, ≥5 weeks old). C57BL/6J (Charles River, France), *NPY-Cre* (Jax #027851), and *Ai14* (Jax #007914) mice were bred in-house after purchasing founders from the original breeding colonies. *NPY-cre:Ai14* were bred in-house. All mice were group housed (two to five per cage) unless otherwise specified. Mice were housed in a 12-hour/12-hour light/dark cycle (lights on at 7:00 a.m.) at 22° ± 2°C (60 to 65% humidity). Unless otherwise specified, animals had access to ad libitum water and lab chow. Experiments were approved by the Animal Ethics Committee of Utrecht University and the Dutch Central Authority for Scientific Procedures on Animals (CCD) and were conducted in agreement with the Dutch law (Wet op de Dierproeven, 2014) and European regulations (Guideline 86/609/EEC).

### Stereotactic surgeries

Mice (≥5 weeks at the time of surgery) were anesthetized with ketamine (75 mg/kg, ip; Narketan, Vetoquinol) and dexmedetomidine (1 mg/kg, ip; dexdomitor, Vetoquinol). Lidocaine (0.1 ml; 10% in saline; B. Braun) was injected under the skull skin, and eye ointment cream (CAF, Ceva Sante Animale B.V.) was applied. Animals were then fixed on a stereotactic frame (UNO B.V. model 68U801 or 68U025), where they kept on a heat pad (33°C) during surgery. For cannula implantations, the skull surface was scratched with a scalpel and phosphoric acid (167-CE, ultra-Etch, ultradent, USA) was applied for 5 min to roughen the surface at the start of surgery. Viral infusions were done using a 31-gauge metal needle (Coopers Needleworks) attached to a 10-μl Hamilton syringe (model 801RN) via flexible tubing [PE10, 0.28-mm inside diameter (ID), 0.61-mm outer diameter (OD), Portex]. The Hamilton syringe was controlled by an automated pump (UNO B.V., model 220). Injections were done bilaterally at 250 nl per side, at an injection rate of 100 nl/min. The injector was gradually retracted during the last minute of a 10-min-long diffusion period. Next, the skin was sutured (V926H, 6/0, VICRYL, Ethicon) and animals were administered the dexmedetomidine antagonist atipamezole [50 mg/kg, subcutaneously (sc); Atipam, Dechra], carprofen (5 mg/kg, sc; Carporal) and 1 ml of saline (sc). Mice were left to recover on a heat plate (36°C) while being monitored and moved to the housing stables when fully awake. Carprofen (0.025 mg/liter) was provided in the drinking water during the first postoperative week. Postsurgery, animals were single housed before rejoining their cagemates at 3 days postoperative. Animals with cannulas were single housed for the remainder of the experiment.

For ex vivo optogenetic or chemogenetic-assisted electrophysiology experiments and ELISA, *NPY-cre* mice were bilaterally injected with rAAV5-Syn-FLEX-CoChR-GFP [4.4 × 10^12^ genome copies (gc) /ml; UNC Vector Core] or rAAV5-hSyn-DIO-hM3D(Gq)-mCherry (4.2 × 10^12^ gc/ml; Addgene) in the LC (AP: −5.45 mm; ML: ± 1.59 mm; DV: −3.96 mm from bregma) under a 10° angle. All animals were allowed to recover for a minimum of 4 weeks before brain collection for electrophysiological recordings and other biochemical assays.

For in vivo chemogenetic experiments, *NPY-cre* mice were bilaterally injected with rAAV5-hSyn-DIO-hM3D(Gq)-mCherry, AAV5-hSyn-DIO-hM4D(Gi)-mCherry, or AAV5-hSyn-DIO-mCherry (all viruses: 4.2 × 10^12^ gc/ml; Addgene) in the LC (AP: −5.45 mm; ML: ± 1.59 mm; DV: −3.96 mm or AP: −5.3 mm; ML: ± 1.95 mm; DV: 3.5 mm from bregma) under a 10° angle. All animals were allowed to recover for 5 weeks before participating in behavioral assays.

For cannula implantations, *NPY-cre* mice were bilaterally implanted with a guide cannula (4 mm; C315GMN/Spc; Bilaney) above the LC (AP: −5.35 mm; ML: ± 1.63 mm; DV: −3.76 mm or AP: −5.35 mm; ML: ± 1.77 mm; DV: −3.86 mm from bregma) under a 10° angle. The cannulas were secured by adding a layer of adhesive luting cement (C&B Metabond; Parkell, Edgewood, NY, USA) around them. Viral infusions were performed immediately after cannula placement by inserting the injector through the guide and lowering it to DV: −3.96 mm. After infusions, dummy internal injectors (4 mm; C315FD/Spc) were locked onto the guides to avoid blockage and remained there until experiment completion. All animals were allowed to recover for 5 weeks before participating in behavioral assays.

For ex vivo biosensor pharmacology or chemogenetic recordings in acute slices, we bilaterally injected a viral cocktail (300 μl) containing AAV9-hSyn-NPY1.0 (3.5 × 10^13^ gc/ml) and rAAV5-hSyn-DIO-hM3D(Gq)-mCherry (4.2 × 10^12^ gc/ml) in the LC (AP: −5.45 mm; ML: +1.59 mm; DV: −3.96 mm, 10° angle) of *NPY*-cre mice.

For in vivo fiber photometry, we unilaterally injected the GRAB_NPY_ sensor (250 μl, 4 × 10^13^ gc/ml) or a GFP-control construct (AAV5-hSyn-GFP; 1.28 × 10^11^ gc/ml) in the LC of C57BL/6 mice. We then inserted an optical fiber (ø400 μm, Thorlabs) 0.16 mm above the injection site (DV: −3.70). The fiber was fixated with superbond glue (Sun Medical Co.), three screws, and dental cement (Fuji PLUS-capsules, G.C. Corporation). After the surgery, the mice were individually housed to prevent fiber damage. All animals were allowed to recover for 5 weeks before participating in behavioral assays.

### Behavioral assays

All behavioral manipulations and tests were performed during the light phase, between noon and 6:00 p.m. Only male mice were included in the behavioral assays described below. Animals were transported to the behavioral room at least 2 weeks before the start of the experiment for acclimatization. Real-time data collection and offline analysis for EPM and NSF tasks were done with Ethovision video tracking (version XT 9 or 11; Noldus, NL).

#### FS stress

Exposure to FS stress was conducted in operant chambers (30 cm by 25 cm by 19 cm, Med Associates) modified for FS delivery, via the grid floor. The chambers were contained in a soundproofing box. Mice were placed in the chamber and given 5 min of habituation to the novel environment. A total of 20 FS (2-s duration, 0.3-mA intensity) were delivered with an interval of 60 s for the next 20 min of the session. The last 5 min of the session (30 min of total duration) were shock-free. Control (no-stress) mice were exposed to the operant chamber (novel environment) in the absence of electrical FS. After the session, all mice were transferred back to their home cage and returned to a holding room until further behavioral assessment.

#### Open field

The open field task was performed using a standard arena (round, diameter of 80 cm). Light intensity in the center of the arena was 25 lux. At the start of the session, mice were placed next to the walls of the arena and allowed to freely explore for 10 min. Behavior was scored for the following variables: time spent in the center or wall zone (% of total exploration time), frequency of visits to the center or wall zone, and total distance moved. The open field arena was cleaned with 70% ethanol solution in-between animals.

#### Elevated plus maze

The EPM task was performed using a standard apparatus (arm length: 65 cm by 65 cm) elevated at 65 cm above ground, equipped with 15-cm-high walls delimiting the enclosed arms. Light intensity in the center of the maze was 40 lux. At the start of the session, mice were placed at one of the closed arms, facing the EPM center, and allowed to freely explore the maze for 10 min. Behavior was scored for the following variables in the first 5 min of the EPM (*83*): time spent in the open or closed arms (OA or CA, respectively; % of total exploration time), frequency of visits to the open or closed arms, total distance moved, and anxiety index (*59*), calculated as: 1 − {[time in OA/(time in OA + time in CA)] + [entries to OA/(entries to OA + entries to CA)]}/2, where values closer to 1 delineate increased anxiety. The maze was cleaned with 70% ethanol solution in-between animals.

Poststress EPM performance (see below) was assessed 4 weeks following a first EPM test to avoid one-trial tolerance, as shown before (*84*). In addition, to prevent habituation to the EPM arena that could confound task performance, the maze was rotated 90°, so as the orientation of closed/open arms was reversed, compared to the first test.

#### Novelty-suppressed feeding

The NSF task was performed using the open field arena as described above. A metal disk containing standard lab chow (one pellet) was firmly affixed at the center of the arena. The pellet itself was attached to the disk, so that the animals could not move it during the test. At the start of the session, mice were placed next to the walls of the arena and allowed to freely explore for 10 min. Behavior was scored for the following variables: time spent in the center or wall zone (% of total exploration time), frequency of visits to the center or wall zone, and total distance moved. In addition, latency to initiate feeding (s) and food consumption in the arena, calculated as: food pellet weight at start − end of session/mouse weight (g/kg), were recorded. A different disk and chow pellet were used per mouse, and the NSF arena was cleaned with 70% ethanol solution in-between animals.

### In vivo chemogenetic experiments

All animals were habituated to restrain for intraperitoneal injections with at least 3x handling sessions that included “sham” injections before the start of the experiments. For intracranial infusions, mice were extensively handled for at least a week (1× day) for familiarization with infusion procedures (dummy removal, injector/tubing plug-in), before the start of the experiments. For all experiments, only mice with confirmed viral expression within the LC and, when applicable, correct cannula or optical fiber placement were included in data analysis. For experiments with chemogenetic manipulations under naïve conditions, seven mice were excluded based on mistargeted virus. For the experiments with intra-LC cannula, 18 mice were excluded, based on misplaced cannula, mistargeted virus, or blocked cannulas during infusion. For experiments with stressed mice and chemogenetic targeting, eight mice were excluded based on mistargeted virus.

For peri-LC_NPY_ stimulation experiments, two separate batches of *NPY-cre* mice were used, with 2 months interval between experiments. Both cohorts (Batch 1: mCherry, *N* = 11; hM3Dq, *N* = 11; Batch 2: mCherry, *N* = 10; hM3Dq, *N* = 10) displayed similar performance; thus, data were pooled.

For intra-LC Y1 antagonism, two separate batches of *NPY-cre* mice were used, with 2.5 months interval between experiments. Both cohorts (Batch 1: mCherry + veh, *N* = 4; hM3Dq + veh, *N* = 5; hM3Dq + BIBO, *N* = 5; Batch 2: mCherry + veh, *N* = 6; hM3Dq + veh, *N* = 5; hM3Dq + BIBO, *N* = 3) displayed similar performance; thus, data were pooled.

#### Elevated plus maze

For peri-LC_NPY_ stimulation or inhibition experiments under naïve conditions, all mice were administered C21 (2 mg/kg, ip). One hour after injection, animals were exposed to a novel environment (MED apparatus, as described above) for 30 min, before being transferred to a clean cage, where they remain single housed for the rest of the experimental procedures. Thirty minutes later, mice were subjected to the EPM test, before returning to their home cage.

For intra-LC Y1 antagonism experiments, internal guides (1-mm projection; C315IMN/Spc; Bilaney) attached to tubing (PE10, 0.28-mm ID, 0.61-mm OD, Portex) were bilaterally inserted in the cannulas. The tubing was attached to a 10-μl Hamilton syringe (model 801RN), controlled by an automated pump (UNO B.V., model 220). While the animals were in their home cage, 200 pM BIBO-3304 or vehicle [1% dimethyl sulfoxide (DMSO) and 99% phosphate-buffered saline (PBS)] was infused in a volume of 200 nl in each hemisphere, at a rate of 100 nl/min. The injector was left in place for an additional minute. Twenty minutes after intracannula infusions, animals were administered C21 (2 mg/kg, ip). Thirty-five minutes after C21 injections, mice were subjected to the EPM task, as described above.

For peri-LC_NPY_ stimulation after stress, mice were subjected to the FS stress paradigm as described above, before returning to their home cage. Twenty-four hours later, animals were administered C21 (2 mg/kg, ip) and participated in the EPM test, 1 hour postinjection.

#### Novelty-suppressed feeding

For peri-LC_NPY_ stimulation or inhibition experiments, mice were subjected to the NSF task, 48 hours after participating in the EPM and following 24 hours of food deprivation. All mice were administered C21 (2 mg/kg, ip), and NSF took place 1 hour postinjection. Immediately after the end of the session, mice were returned to their home cage, where food (standard laboratory chow) intake was recorded for 5 min.

#### Ad lib food consumption

Mice with ad libitum food availability were administered C21 (2 mg/kg, ip) and left undisturbed in their home cage. Food consumption (standard laboratory chow) was recorded 1 hour postinjection, for a total of 3 hours, calculated as: food weight at start − end of session/mouse weight (g/kg).

### Patch-clamp electrophysiology

Animals were anesthetized with pentobarbital (Euthasol, 20%, 0.1 ml, ip) between 8:30 and 10:00 a.m. and transcardially perfused with ice-cold carbogenated (95% O_2_ and 5% CO_2_) slicing solution containing 92 mM choline chloride, 10 mM ascorbic acid, 0.5 mM CaCl_2_, 25 mM glucose, 20 mM Hepes, 2.5 mM KCl, 3.1 mM *N*-acetyl-l-cysteine, 25 mM NaHCO_3_, 1.2 mM NaH_2_PO_4_, 29 mM *N*-methyl-d-glucamine (NMDG), 7 mM MgCl_2_, 3 mM sodium pyruvate, and 2 mM thiourea. Brains were quickly extracted, placed on a vibratome (1200 VTs, Leica) and sliced in the coronal plane at 250-μm thickness, in ice-cold slicing solution. Slices recovered for 30 min at 36°C in carbogenated solution of identical composition. Thereafter, slices were maintained at room temperature (RT) in carbogenated incubation solution containing 3 mM ascorbic acid, 2 mM CaCl_2_, 25 mM glucose, 20 mM Hepes, 2.5 mM KCl, 92 mM NaCl, 20 mM NaHCO_3_, 1.2 mM NaH_2_PO_4_, 29 mM NMDG, 2 mM MgCl_2_, 3 mM sodium pyruvate, and 2 mM thiourea. During recordings, slices were immersed in artificial cerebrospinal fluid (ACSF) containing 2.5 mM CaCl_2_, 11 mM glucose, 5 mM Hepes, 2.5 mM KCl, 124 mM NaCl, 26 mM NaHCO_3_, 1 mM NaH_2_PO_4_, and 1.3 mM MgCl_2_ and were continuously superfused at a flow rate of 2 ml min^−1^ at 28° to 30°C.

Peri-LC_NPY_ or LC_NE_ neurons were patch-clamped using borosilicate glass pipettes (2.7 to 4.5 megohms; glass capillaries, GC150-10, Harvard apparatus, UK), under a TH4-200 Olympus microscope (Olympus, France). For voltage- or current-clamp recordings, signals were amplified and digitized using a HEKA EPC-10 patch-clamp amplifier (HEKA Elektronik GmbH). Data were acquired using PatchMaster v2x90.2 software. Access resistance was monitored with a −4-mV step delivered at 0.1 Hz. Experiments were discarded if the access resistance increased by more than 20% during the recording. All electrophysiological measures are recorded with a 10-s intersweep interval (0.1 Hz).

#### Intrinsic excitability

Recordings were made in a Kglu-based internal containing 139 mM Kglu, 10 mM Hepes, 0.2 mM EGTA, 10 mM creatine phosphate, 5 mM KCl, 4 mM Na_2_ATP, 0.3 mM Na_3_GTP, and 2 mM MgCl_2_. Upon break-in, cells were kept at −50 mV for 10 min prior to the onset of current-clamp recordings. To assess passive membrane properties and cell firing patterns, neurons were subjected to 17 consecutive current steps of 800-ms length, starting from −150 to +250 pA, with a 25-pA interstep increment.

For further validation of the cellular identity of putative LC_NE_ neurons, LC-containing slices were obtained from C57BL/6 mice. Recordings were made in continuous perfusion of ACSF before and after clonidine (1 μM) addition. Spontaneous firing (0-pA current step) was sampled for a minimum of 12 sweeps x 800-ms length, with a 30-s intersweep interval, until stable baseline (~1-Hz firing frequency) was reached. Clonidine effects were measured ~3 min after bath application and for an average of five sweeps.

For stress effects on peri-LC_NPY_ or LC_NE_ excitability, LC-containing slices from *NPY-cre:Ai14* mice were obtained 30 min after FS stress or exposure to the novel environment (no-stress controls). LC_NPY_ cells were identified by tdTomato expression under the microscope. LC_NE_ neurons were identified based on location and morphological characteristics.

For ex vivo chemogenetic experiments, effects of bath-applied C21 were examined in LC-containing slices from *NPY-cre* mice at ≥5 weeks after DIO-hM3D(Gq) injection. Recordings were made in continuous perfusion of C21 (2 μM) or vehicle (0.1% DMSO). When applicable, slices were pretreated with synaptic blockers (10 μM CNQX, 50 μM D-AP5, 100 μM picrotoxin, and 10 μM CGP-54626), Y1R (1 μM BIBO-3304), or Y2R (1 μM BIIE-0246) antagonists for 10 min before being transferred in ACSF containing C21 or vehicle and the correspondent antagonist mix. All slices for chemogenetic experiments were controlled for virus expression at the end of recordings. Only data from slices with strong hM3D(Gq) expression within the peri-LC were taken along for analysis.

For NPY effects on LC_NE_ excitability, LC-containing slices were obtained from wild-type C57BL/6 mice. Recordings were made in continuous perfusion of NPY (30 or 300 nM) or vehicle (0.1% DMSO). When applicable, slices were pretreated (10 min) with synaptic blockers as described above, before being transferred in ACSF containing NPY or vehicle.

For validation of DREADD constructs, effects of bath-applied C21 were examined in LC-containing slices from *NPY-cre* mice at ≥5 weeks after DIO-hM3D(Gq) or DIO-hM4D(Gi) injection. Peri-LC_NPY_ cells were identified by mCherry expression under the microscope. Peri-LC_NPY_ spontaneous activity was recorded before and after C21 (2 μM) bath application.

#### Peri-LC_NPY_ to LC_NE_ optogenetic synaptic connectivity experiments

At ≥5 weeks after virus injection of FLEX-CoChR in the LC of *NPY-cre* mice, recordings were made in voltage clamp in a Kglu-based internal solution containing 139 mM Kglu, 10 mM Hepes, 0.2 mM EGTA, 10 mM creatine phosphate, 5 mM KCl, 4 mM Na_2_ATP, 0.3 mM Na_3_GTP, and 2 mM MgCl_2_ at −50 mV for the detection of inward glutamatergic and outward GABAergic currents. Alternatively, recordings were performed with a CsCl-based internal solution containing 139 mM CsCl, 10 mM Hepes, 0.2 mM EGTA, 10 mM creatine phosphate, 5 mM NaCl, 4 mM Na_2_ATP, 0.3 mM Na_3_GTP, 2 mM MgCl_2_, and 0.1 mM spermine, at −60 mV or +40 mV for detecting inward GABA-mediated or outward NMDA-mediated currents, in correspondence. Responses to single optical pulse (470 nm, 1 ms, 1 to 2 mW) or trains of 5, 20, and 50 Hz delivered through the light path of the microscope powered by a light-emitting diode (LED) driver (LEDD1B; Thorlabs, Newton, NJ) were recorded. Connectivity was determined based on whether a neuron showed a photoevoked synaptic response of ≥5 pA, over an average of 10 to 20 sweeps. In a portion of recordings, internal solutions contained 3% biocytin (B4261, Sigma-Aldrich), for cell-filling and post hoc identification. All slices for optogenetic experiments were controlled for virus expression at the end of recordings. Only data from slices with CoChR expression within the peri-LC were included in the connectivity analysis.

#### Spontaneous inhibitory postsynaptic currents

LC-containing acute slices were collected, and recordings were made in voltage clamp in a CsCl-based internal medium as described above. Spontaneous inhibitory postsynaptic currents (sIPSCs) onto LC_NE_ neurons were recorded at −60 mV, in the presence of the AMPA/kainate receptor antagonist CNQX (10 μM). To establish stable measurements, recordings of 30 min per cell were performed. Spontaneous events were detected and analyzed in Mini-Analysis (Synaptosoft). Data presented (IPSC frequency and amplitude) are averaged over the last 5 min of the recordings.

### GRAB_NPY_ imaging in brain slices

At >12 weeks after virus injection, LC-containing slices from wild-type C57BL/6 (pharmacological validation of the GRAB_NPY_ sensor) or *NPY-cre* mice (chemogenetically evoked NPY release) were collected as describe before for patch-clamp electrophysiology. The (peri-)LC was identified using a back-illuminated scientific complementary metal-oxide-semiconductor (sCMOS) camera (5.76-megapixel, 6.5-μm by 6.5-μm pixel size, 16-bit, 22-mm sensor diagonal, Kinetix 22; Teledyne Photometrics AZ, USA), mounted on an Axio Examiner A1 microscope (Zeiss), using a 40x objective (W N-Achroplan 40x/0.75 M27; Zeiss). Data were acquired using the Micro-Manager 2.0.3 software, packaged as an ImageJ plug-in. Fluorescent images were taken after delivery of 10-ms pulses of max 0.4-mW intensity, driven by a 450-nm LED (pE-400, CoolLed), at a sampling interval of 1 s, using a dual-pass filter set (including 450- to 490-nm excitation and 499- to 545-nm emission wavelengths; SCI-59022s; Scientifica). Slices were continuously perfused, at RT, with ASCF (0.1% DMSO) for a minimum of 5 min of baseline, followed by application of NPY (300 nM) or of C21 (2 μM). Data were analyzed using FIJI (*85*), by drawing a region of interest (ROI) containing the LC and pericoerulean space and then plotting *z*-axis profile of the entire image stack (*z* series of 600 images per 5 min of recordings). Pixel intensity change (∆*F*/*F*) was calculated relative to the average baseline signal (sampling values at −4 to 0 min), from NPY or C21 superfusion (0 min) onward, in 4-min time bins.

### Fiber photometry recordings with the GRAB_NPY_ sensor

Mice were subjected to the FS stress paradigm (see above) while being recorded with a two-site photometry system of Doric lenses consisting of a console (FPC), LED driver (LEDD_2), and two fluorescent mini cubes [ilFMC5-G2_IE(400-410)_E(460-490)_F(500-540)_O(580-680)_S], recording 227 samples/s (Doric Studio, version 6.1.1.4.). Blue (465 nm, 40 ± 5 μW) and purple (405 nm, 20 ± 3 μW) LED light was delivered in the brain via a mini cube and patch cord (ø400 μm). The emitted light was detected (ac mode, Doric detector) and transmitted to the console with reference frequencies of 572.2 Hz for the blue (GFP) and 333.8 Hz for the isosbestic (non-GFP, control) channel. The relative light change (∆*F*/*F*) time-locked to shock delivery was calculated with custom-made Python scripts (version 3.1.1). First, a Butterworth low-pass filter (6 Hz) was applied, the autofluorescence was subtracted, and the data were smoothed (per 50 data points) from the raw signal. We calculated the ∆*F*/*F* of each shock relative to the average baseline signal of the 5 s before shock onset.

### Histology and immunolabeling

All mice were anesthetized with pentobarbital (Euthasol, 0.1 ml, ip) and transcardially perfused with PBS followed by freshly made ice-cold 4% paraformaldehyde (PFA). Brains were extracted and postfixated in 4% PFA overnight, before being transferred to an antifreeze solution (30% sucrose) until they sank. Thereafter, sections measuring 35 μm (colocalization studies) or 50 μm (histological control of virus targeting) were collected using a cryostat (Leica CM 1950) at −12°C and stored in PBS and 0.01% NaN_3_ until Immunolabeling. Brains from animals participating in cannula and fiber photometry experiments were postfixated for 48 hours and transferred to 10% sucrose before being embedded to a 10% sucrose/10% gelatin solution. Next, embedded brains were refixated in 10% sucrose/4% PFA solution overnight and stored in 30% sucrose until slicing. Sections (50 μm) were collected with a vibrotome (Leica VT 1000S) at RT. In most cases, every fifth consecutive section was collected for further processing to ensure LC representation in the entire rostrocaudal axis. For immunohistochemical stainings, sections were washed in 1× PBS and blocked (1 hour, RT) in a solution containing 5% normal goat or donkey serum, 2.5% bovine serum albumin (BSA), and 0.2% Triton X-100. Primary antibodies against NPY (Novus Biologicals, NBP1-46535), TH (LNC1, Millipore, MAB318), GABA (Sigma-Aldrich, A2052), SST (OriGene, AP33464SU-N), ChAT (Millipore, AB144P), cFos (9F6, Cell Signaling Technology, 2250S), and GFP (Aves, GFP-1020) were incubated overnight at 4°C. Secondary antibodies (Alexa Fluor 488, 568, or 647, Invitrogen) were incubated for 2 hours (RT), in some cases, followed by 4′,6-diamidino-2-phenylindole (DAPI) nuclear staining (20 min, RT). Slices were mounted in 0.2% gelatin and coverslipped with DABCO antifading medium (Merck, 10981).

For NPY topography studies, sections were imaged at 10x magnification using a confocal microscope (LSM 880, Zeiss). Tiling was performed to include the ML space to LC, up to 1 mm from the LC proper. Images were processed with FIJI, and an in-house macro was used for detection of tdT^+^ and TH^+^ cells. Location of NPY^+^ neurons was determined based on *X* (ML) and *Y* (DV) coordinates as calculated against an ROI representing the LC center of mass per image. Frequency distributions for NPY^+^ cell location in ML and DV axes were calculated for three different rostrocaudal ranges, namely, 5.80 to −5.60 mm, −5.60 to −5.40 mm, and −5.40 to −5.25 mm, and averaged over the corresponding images.

For colocalization studies, sections were imaged by confocal microscope at 20× or 40× magnification and *z*-stacks (≥8 images) were processed with FIJI. An in-house macro was used for detection of tdT^+^, GABA^+^, SST^+^, ChAT^+^, TH^+^, and cFos^+^ cells, which overlaid detected ROIs in one channel over the second. For quantification of tdT^+^/GABA^+^ double-expressing neurons, the following inclusion criteria were used: (i) GABA channel intensity ≥ 66% of a positive, GABA-expressing “example” cell and (ii) GABA channel intensity ≥ 2× of the background. For quantification of tdT^+^/cFos^+^ double-expressing cells, the following inclusion criteria were used: (i) cFos channel intensity ≥ 50% of a positive, cFos-expressing “example” cell and (ii) cFos channel intensity ≥ 150% of the background, per image.

### RNAscope, ViewRNA ISH assays, and analysis

ISH was performed as per the manufacturer’s instructions (ACDBio, RNAscope Multiplex Fluorescent Reagent kit v2, 317621; ViewRNA Tissue Assay Fluorescence Tissue Fluor. 4 Plex Assay Kit, Thermo Fisher Scientific, QVT4700). Briefly, animals were transcardially perfused with sterile 4% PFA and postfixated overnight at 4°C. Brains were transferred to 10% sucrose until they sank. This step was repeated with 20 and 30% sucrose. Brains were then embedded in optimal cutting temperature media (Tissue-Tek, VWR, NL) and placed in the cryostat at −20°C for 1 hour to equilibrate. Sections measuring 10 μm (RNAscope) or 20 μm (ViewRNA) containing the LC were then mounted on SuperFrost Plus slides (VWR, NL) and allowed to dry at −20°C for 2 hours. For RNAscope ISH, sections were pretreated with hydrogen peroxide for 10 min before target retrieval at 99°C for 5 min and treatment with Protease III for 30 min at 40°C. Hybridization to probes against *Npy* (313321), *Npy1r* (427021), *Npy2r* (315951), or *Th* (317621) was carried out at 40°C for 2 hours. Horseradish peroxidase (HRP) signals against each channel (C1 to C3) were then sequentially amplified and developed using TSA Vivid fluorophores (520, 570, and 650) at a dilution of 1:1500 or 1:3000. Positive (3-plex PN 320881) and negative (3-plex PN 320871) control probes were included in each experiment to assess sample RNA quality and optimal permeabilization conditions. In some cases, immunolabeling was combined with ISH. For this, sections were blocked with 0.1% BSA in tris-buffered saline (30 min, RT) before incubation of primary antibody against TH (1.5 hours, RT). Sequential secondary HRP (30 min, RT) and TSA (10 min, RT) incubations were performed next. Sections were counterstained with DAPI for 30 s, coverslipped with ProLong Gold Antifade Mountant, and allowed to dry overnight at RT. For ViewRNA ISH, slides were dried 1 hour prior to transfer in PBS, followed by Protease QF treatment for 10 min at 40°C. Sections were fixed with freshly prepared 4% PFA for 10 min at RT. Hybridization to probes against *Th* (VB4-3112458-VC), *Slc17a6* (VB1-17688-VC), *Npy* (VB1-14823-VC), and *Gad2* (VB10-3282565-VC) was carried out at 40°C for 4 hours. Incubation time and temperature were preoptimized for optimal signal and tissue integrity. Signals were detected using probes conjugated to Alexa Fluor 488/568/657/750, as indicated by the manufacturer, and counterstained with DAPI (1 μg/ml) for 10 min. Slides were embedded in FluorSave Reagent (Merck Millipore, #345789) and allowed to dry 1 hour at RT and overnight at 40°C. An ACDBio hybridization oven was used for all incubation steps for both in situ protocols. Confocal mages (20× or 40× magnification) were processed with FIJI and an in-house macro for detection of *Npy*-, *Npy1r*-, *Npy2r*-, *Slc17a6*-, *Gad2*-, or *Th*-expressing cells.

### Enzyme-linked immunosorbent assay

For ELISA experiments, DIO-hM3D(Gq)–expressing *NPY-cre* mice were used as described above. LC-containing slices we collected as described for electrophysiological (patch-clamp) experiments. Five biological replicates per treatment (ASCF or C21) group were prepared as follows: Per replicate, slices (~4 per animal) from four mice were pooled together in one well of a 12-well cell culture plate containing 1 ml of ACSF. Five minutes later, the supernatant was collected and the medium was replaced with 1 ml of either ACSF or C21 (2 μM)–containing ASCF. Twenty minutes later, the supernatant was again collected and flash frozen in dry ice. Supernatant samples were kept in −20°C until further use. First, a BCA assay (Pierce BCA Protein Assay Kits, Thermo Fisher Scientific, A55865) was performed to determine total protein concentration per sample, according to the manufacturer’s guidelines. Then, samples (5 μg of protein) were loaded in an NPY ELISA kit (Antibodies Online, ABIN6968884) and the NPY concentration was determined against a standard of serial dilutions, according to the manufacturer’s instructions. In brief, diluted standard and samples were loaded in replicate in an NPY precoated 96-well plate and incubated for 90 min at 37°C. After washing, wells were treated with a biotin-labeled antibody and incubated for 60 min at 37°C. After washing, wells were treated with an HRP-streptavidin conjugate and incubated for 30 min at 37°C. After a final incubation with a 3,3′,5,5′-tetramethylbenzidine (TMB) substrate (15 min at 37°C), the plate was scanned using a Varioskan LUX Multimode Microplate Reader (Thermo Fisher Scientific, USA). Optical density absorbance was read at 450 nm, and the NPY concentration per sample was interpolated from the standard curve.

### Data analysis, statistics, and reproducibility

Data were analyzed with Noldus (Ethovision XT V9 or 11), GraphPad Prism (9.5.1), Igor Pro-8 (Wavemetrics, USA), FIJI, Python (v 3.1.1), and IBM SPSS (v 27). Sample size was predetermined on the basis of published studies, experimental pilots, and in-house expertise. Animals were randomly assigned to experimental groups. The number of mice (*N*) and/or cells (*n*) is included in figure legends. Compiled data are always reported and represented as means ± SEM, with single data points plotted (single cell for electrophysiology and single animal for behavioral experiments). Animals or data points were not excluded from analyses unless noted. Statistical comparisons were conducted using paired or unpaired *t* tests and one-way or two-way repeated measure analysis of variance (ANOVA). In the case of significant main (interaction) effects, post hoc comparisons were performed. Normality distribution was confirmed with the Kolmogorov-Smirnov test, and in the case of violation, nonparametric Mann-Whitney or Wilcoxon signed-rank tests were performed. Two-tailed testing was performed with significance level α set at 0.05.
