# Supplementary material for: Neuropeptide Y neurons surrounding the locus coeruleus inhibit noradrenergic system activity to reduce anxiety
Source: Sci Adv. 2025 Jul 23;11(30):eadq0011. doi: 10.1126/sciadv.adq0011 (PMC12285701; doi:10.1126/sciadv.adq0011)
Supplement: Supplementary file 1 — Figs. S1 to S10 Tables S1 and S2 Legend for data S1 [file sciadv.adq0011_sm.pdf]

Science Advances
AAAS

Supplementary Materials for

Neuropeptide Y neurons surrounding the locus coeruleus inhibit noradrenergic system activity to reduce anxiety

Danai Riga *et al.*

Corresponding author: Frank J. Meye, f.j.meye-2@umcutrecht.nl

Sci. Adv. **11**, eadq0011 (2025)
DOI: 10.1126/sciadv.adq0011

The PDF file includes:

Figs. S1 to S10
Tables S1 and S2
Legend for data S1

Other Supplementary Material for this manuscript includes the following:

Data S1

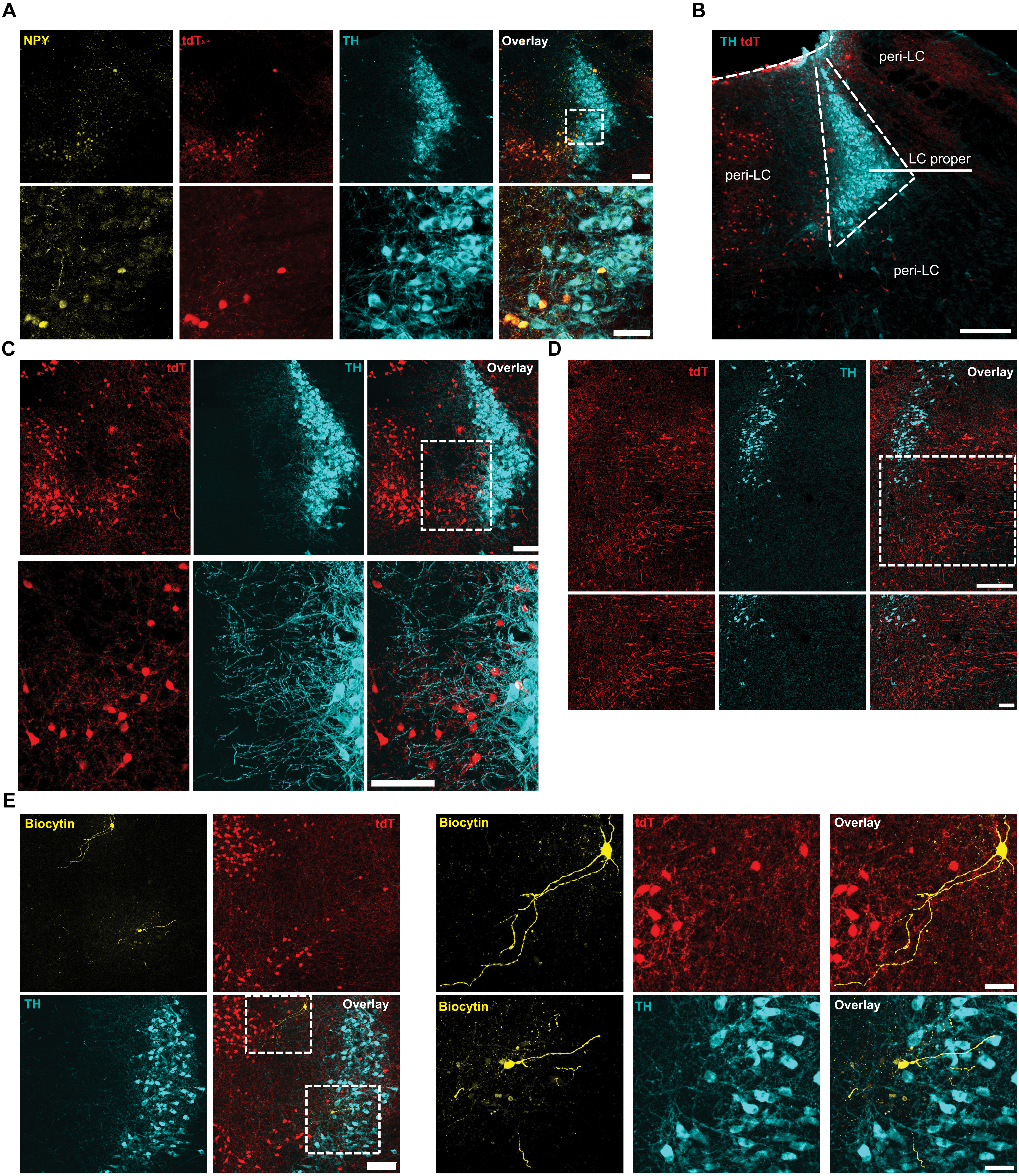
A
NPY
tdT
TH
Overlay
B
TH tdT
peri-LC
LC proper
peri-LC
peri-LC
C
tdT
TH
Overlay
D
tdT
TH
Overlay
E
Biocytin
tdT
TH
Overlay
Biocytin
tdT
Overlay
Biocytin
TH
Overlay

Figure S1

Figure S1. NPY-expressing neurons of the (peri)LC.

A) Representative example of tdTomato expression (tdT, red), NPY (yellow) and LC_{NE} neurons (TH, cyan) immunolabeling in coronal slices from *NPY-cre:Ai14* mice, depicting the LC. Inset: Tdt^{+} neurons colocalize with endogenous NPY, but not TH; Scale bar, 100µm and (inset) 50µm.

B) Representative image of the LC proper, as indicated by TH^{+} (cyan) LC_{NE} cell bodies, and the pericoerulean space occupied by NPY-expressing (tdT^{+}, red) neurons. Scale bar, 200µm.

C) Representative image of NPY-expressing cells (tdT, red) and LC_{NE} neurons (TH, cyan). Peri-LC_{NPY} neurons concentrate medial of the LC nuclear core, at an area where LC_{NE} cells receive the majority of input, known as the LC dendritic zone. Scale bar, 100µm and 50µm (inset).

D) Representative image of NPY-expressing cells (tdT, red) and LC_{NE} neurons (TH, cyan). Peri-LC_{NPY} neurons dendritic fibers occupy the pericoerulean space. Scale bar, 250µm and 100µm (inset).

E) Representative examples of biocytin-filled NPY-expressing cells (yellow) in the LC. Peri-LC_{NPY} neuronal processes extend towards the LC dendritic zone and are intermingled with LC_{NE} cell bodies (TH, cyan). Scale bar, 100µm and 50µm (insets).

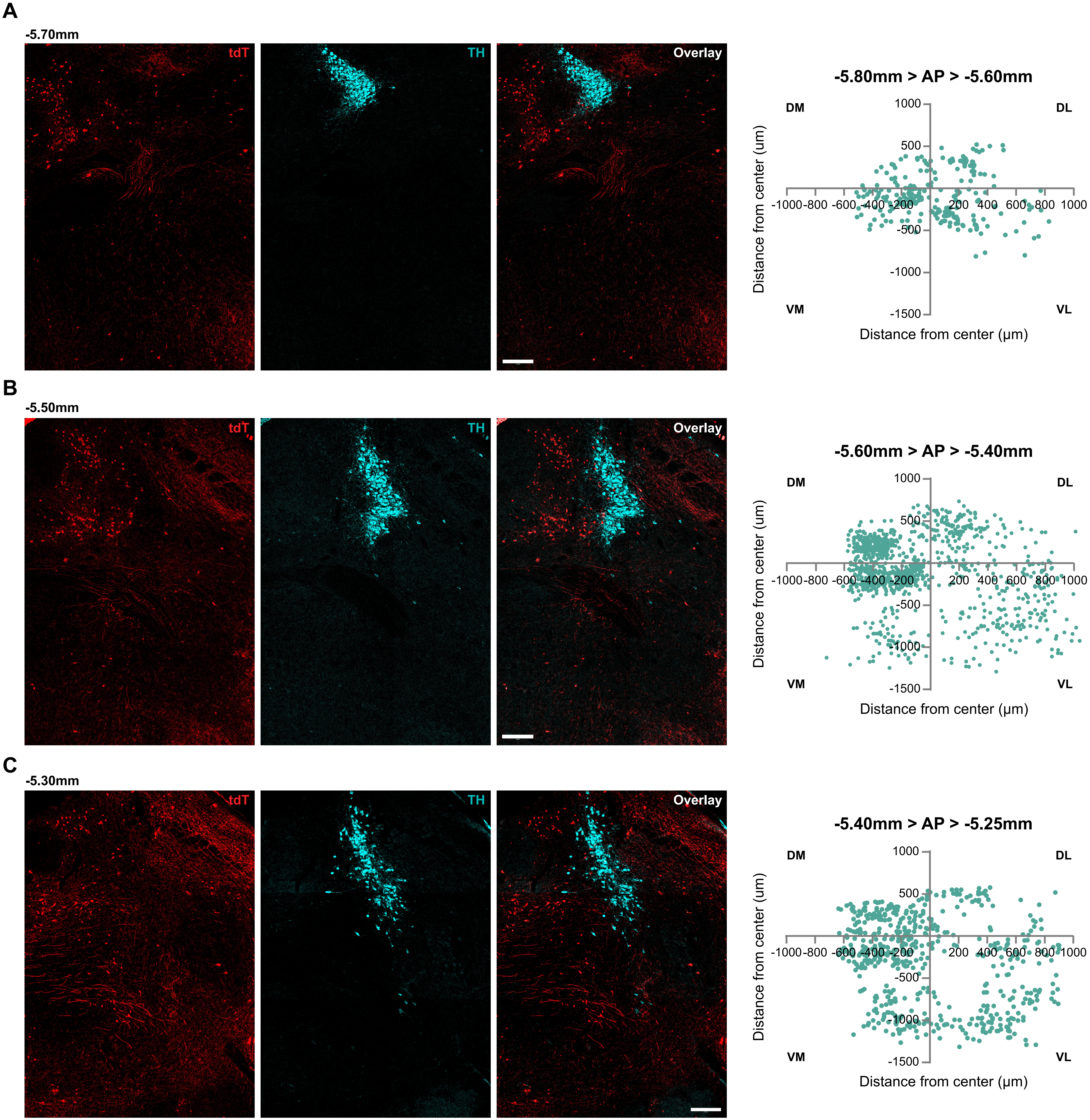
A
-5.70mm
tdT
TH
Overlay
-5.80mm > AP > -5.60mm
B
-5.50mm
tdT
TH
Overlay
-5.60mm > AP > -5.40mm
C
-5.30mm
tdT
TH
Overlay
-5.40mm > AP > -5.25mm
DM
DL
VM
VL
Distance from center (um)
Distance from center (μm)
1000
500
-500
-1000
-1500
-1000 -800 -600 -400 -200 200 400 600 800 1000

Figure S2

Figure S2. Distribution of NPY neurons in the pericoerulean space.

A-C) *Left*: Representative images of the location of peri-LC_{NPY} neurons, identified by tdT expression. *Right*: NPY neuron location was mapped against LC_{NE} cells in the entire rostrocaudal axis containing the LC. Each data point represents a single tdT^+ cell, from which x (mediolateral) and y (dorsoventral) coordinates were extracted and plotted in respect to its distance (µm) from LC center. Data accumulated over three AP ranges. Scale bars, 200µm. DM: dorsomedial, DL: dorsolateral, VM: ventromedial, VL: ventrolateral. AP, -5.80 to -5.60 mm, N=2, n=239; AP, -5.60 to -5.40 mm, N=7, n=1004; AP, -5.40 to -5.25 mm, N=6, n=558.

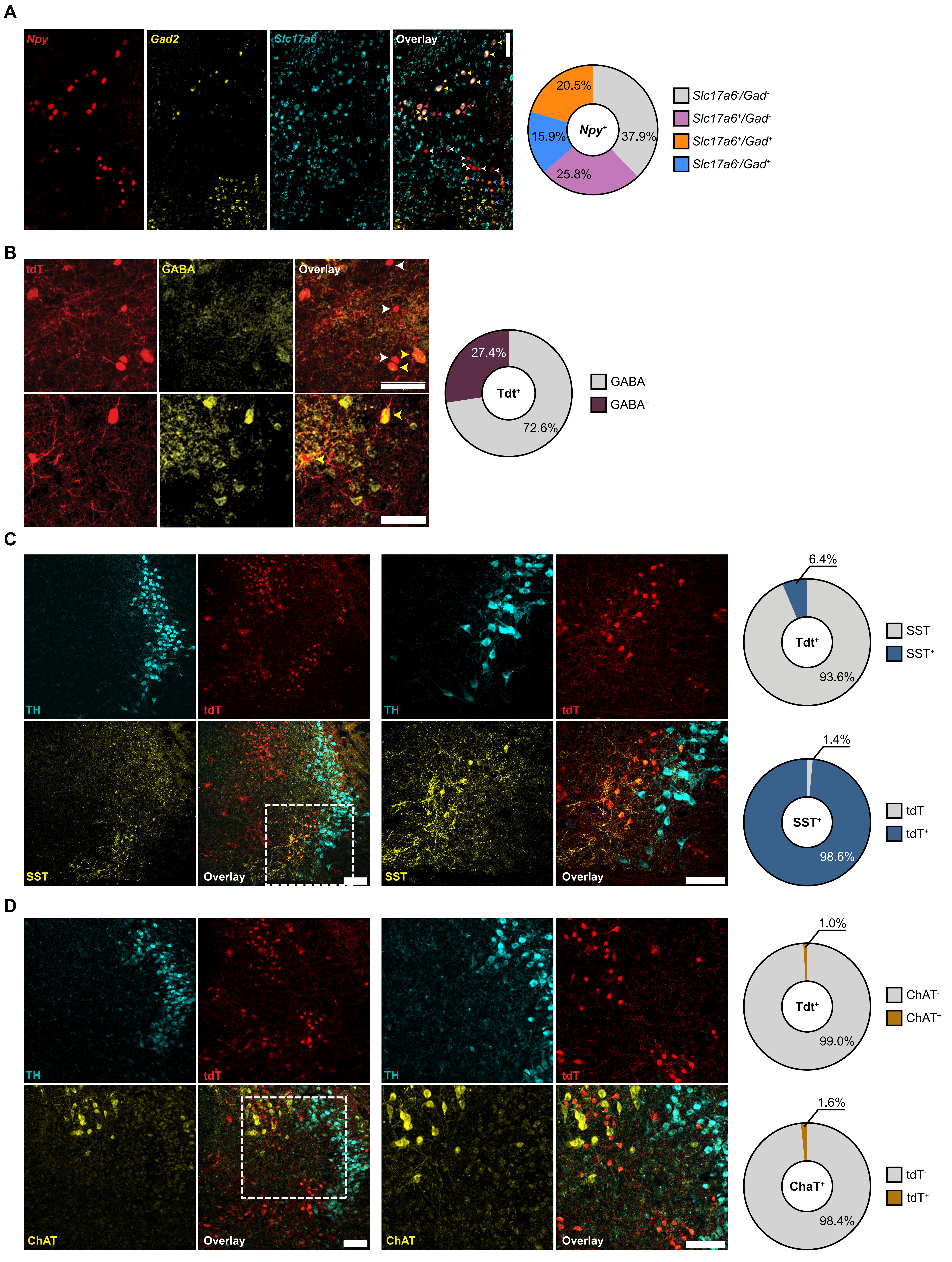
A
Npy
Gad2
Slc17a6
Overlay
20.5%
15.9%
Npy+
37.9%
25.8%
Slc17a6-/Gad-
Slc17a6+/Gad-
Slc17a6+/Gad+
Slc17a6-/Gad+
B
tdT
GABA
Overlay
27.4%
Tdt+
72.6%
GABA-
GABA+
C
TH
tdT
SST
Overlay
6.4%
Tdt+
93.6%
SST-
SST+
1.4%
SST+
98.6%
tdT-
tdT+
D
TH
tdT
ChAT
Overlay
1.0%
Tdt+
99.0%
ChAT-
ChAT+
1.6%
ChaT+
98.4%
tdT-
tdT+

Figure S3

Figure S3. Molecular characterization of peri-LC$_{NPY}$ neurons

A) *Left*: Representative examples of *Npy* (red), *Gad2* (yellow) and *Slc17a6* (cyan) mRNA in coronal slices from C57BL/6 mice. *Right*: Quantification of colocalization. The majority of peri-LC$_{NPY}$ neurons are exclusively peptidergic (*Slc17a6*$^-$/*Gad2*$^-$; 37.9 %, white arrowheads). In addition, we identified glutamatergic (*Slc17a6*$^+$/*Gad2*$^-$; 25.8 %, fuchsia arrowheads), GABAergic (*Slc17a6*$^-$/*Gad2*$^+$; 15.9 %, blue arrowheads) and combinatorial (*Slc17a6*$^+$/*Gad*$^+$; 20.5 %, yellow arrowheads) subpopulations. Scale bar, 100µm. N=5 mice, n=523 cells.

B) *Left*: Representative examples of tdTomato expression (tdT, red) and GABAergic immunolabeling (GABA, yellow) in coronal slices from *NPY-cre:Ai14* mice. *Right*: Quantification of colocalization. Only a subset of peri-LC$_{NPY}$ neurons co-express GABA. GABA$^-$ cells (72.6 %, white arrowheads); GABA$^+$ cells (27.4 %, yellow arrowheads). Scale bar, 50µm. N=7 mice, n=2371 cells.

C) *Left*: Representative examples of tdTomato expression (tdT, red) and somatostatin immunolabeling (SST, yellow) in coronal, LC-containing (TH, cyan) slices from *NPY-cre:Ai14* mice. *Right*: Quantification of colocalization. A small subpopulation of peri-LC$_{NPY}$ neurons co-express SST (SST$^+$, 6.4 %; SST$^-$, 93.6 %). Conversely, the vast majority of peri-LC SST$^+$ cells co-express NPY (tdT$^+$, 98.6 %; tdT$^-$, 1.4 %). Scale bar, 100µm. N=6 mice; tdT$^+$, n=1111 cells; SST$^+$, n=71 cells.

D) *Left*: Representative examples of tdTomato expression (tdT, red) and choline acetyltransferase immunolabeling (ChAT, yellow) in coronal, LC-containing (TH, cyan) slices from *NPY-cre:Ai14* mice. *Right*: Quantification of colocalization. Peri-LC$_{NPY}$ neurons rarely colocalize with ChAT (ChAT$^+$,1.0 %; ChAT$^-$, 99 %). Likewise, peri-LC ChAT$^+$ cells do not co-express NPY (tdT$^+$, 1.6 %; tdT$^-$; 98.4 %). Scale bar, 100µm. N=6 mice. tdT$^+$, n=816 cells; ChAT$^+$, n=507 cells.

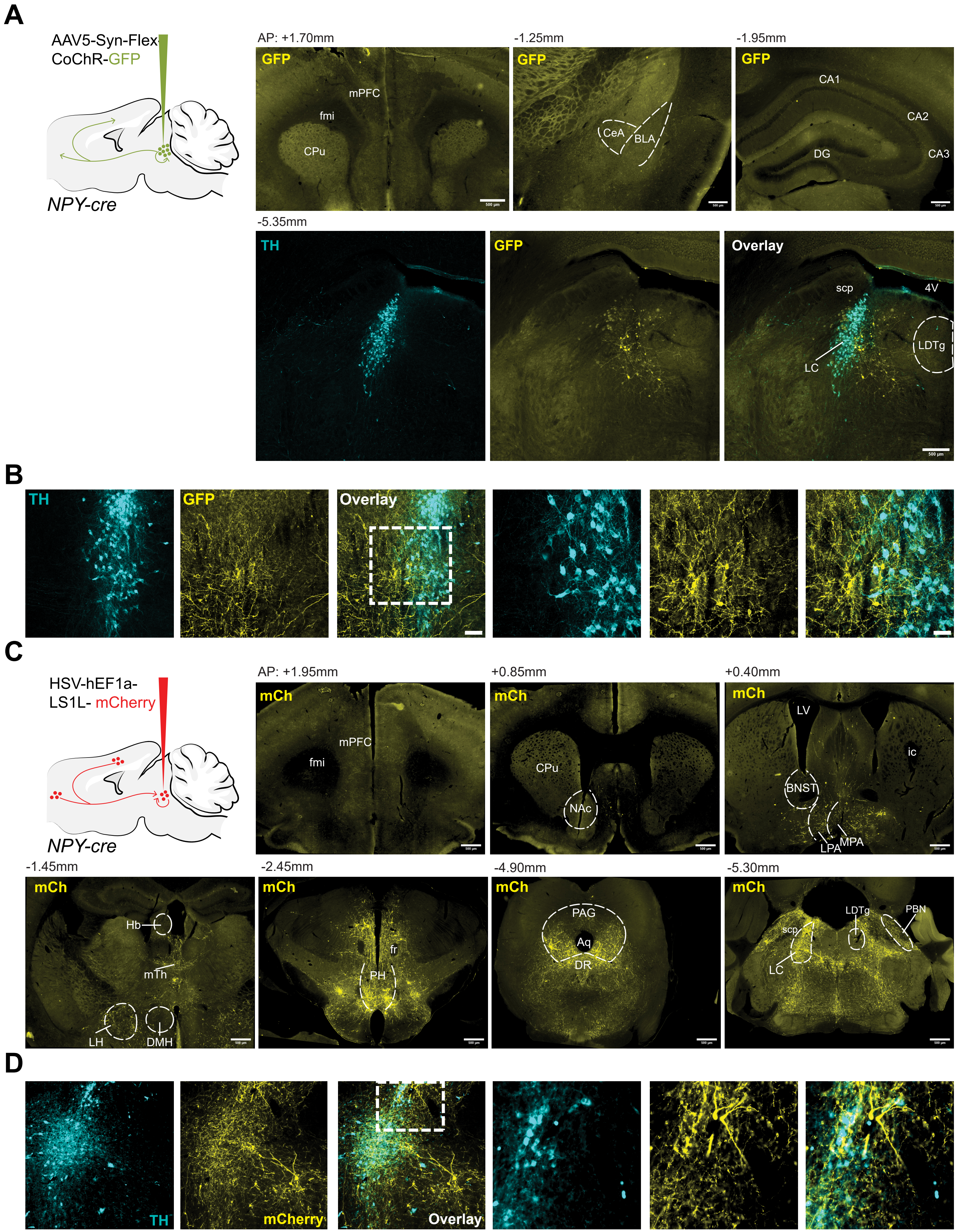
A
AAV5-Syn-Flex-CoChR-GFP
NPY-cre
AP: +1.70mm
-1.25mm
-1.95mm
GFP
mPFC
fmi
CPu
CeA
BLA
CA1
CA2
CA3
DG
-5.35mm
TH
Overlay
scp
4V
LDTg
LC
B
TH
GFP
Overlay
C
HSV-hEF1a-LS1L-mCherry
NPY-cre
AP: +1.95mm
+0.85mm
+0.40mm
mCh
mPFC
fmi
CPu
NAc
LV
ic
BNST
LPA
MPA
-1.45mm
-2.45mm
-4.90mm
-5.30mm
Hb
mTh
LH
DMH
fr
PH
PAG
Aq
DR
scp
LDTg
PBN
LC
D
TH
mCherry
Overlay

Figure S4

Figure S4. Anterograde and retrograde tracing of peri-LC_{NPY} neuroanatomical circuitry

A, B) *Left:* Schematic of sagittal mouse brain, depicting the location of bilateral virus injections used to drive CoChR expression in *NPY-cre* mice. *Right*: Representative images show lack of NPY efferents in known LC projection fields, such as the prefrontal cortex, amygdala and the hippocampus. Conversely, GFP^+ cell bodies and projections are seen in the pericoerulean space (TH, tyrosine hydroxylase). Scale bar, 500 µm N=5.

B)) Representative images of anterogradely labeled GFP^+ cell bodies and projections (yellow) in the LC dendritic zone and among LC_{NE} cells (TH, tyrosine hydroxylase). Scale bar, 100 µm.

C) Schematic of sagittal mouse brain, depicting the location of bilateral virus injections for retrograde labeling in *NPY-cre* mice. Several NPY afferent regions were identified (*cf.*, Table S1), with NPY neurons from hypothalamic regions and the PAG heavily innervating the LC. N=5.

D) Representative images of mCherry expression (yellow) and noradrenergic (TH, cyan) immunolabeling in retrogradely-labelled slices from *NPY-cre* mice. Scale bar, 100µm and 50µm (inset).

Aq, aqueduct; BLA, basolateral amygdala; BNST, bed nucleus of the stria terminalis; CA1, CA2, CA3 hippocampal subfields; CeA, central amygdala; CPu, caudate putamen; DMH, dorsal medial hypothalamic area; fmi, forceps minor of the corpus callosum; fr, fasciculus retroflexus; ic, internal capsule; LC, locus coeruleus; LDTg, laterodorsal tegmental nucleus; LH, lateral hypothalamic area; Hb, habenula; LPA, lateral preoptic area; LV, lateral ventricle; MPA, medial preoptic area; NAc, nucleus accumbens; PAG, periaqueductal gray; DR, dorsal raphe; PBN, parabrachial nucleus; PH, posterior hypothalamic area; mPFCL, medial prefrontal cortex; scp, superior cerebellar peduncle. Scale bar, 500µm.

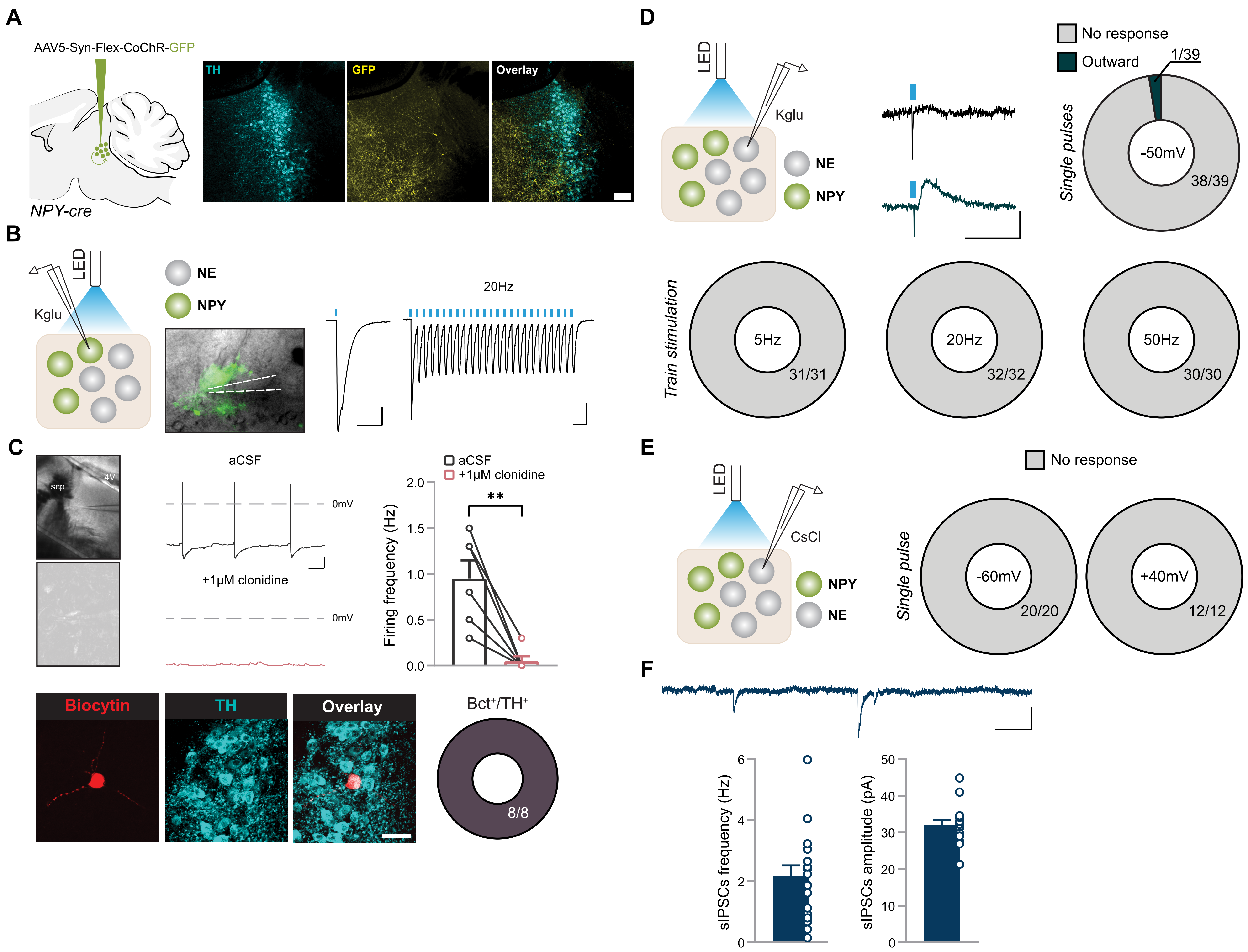
A
AAV5-Syn-Flex-CoChR-GFP
NPY-cre
TH
GFP
Overlay
B
LED
Kglu
NE
NPY
20Hz
C
scp
4V
aCSF
0mV
+1µM clonidine
0mV
aCSF
+1µM clonidine
**
Firing frequency (Hz)
2.0
1.5
1.0
0.5
0.0
Biocytin
TH
Overlay
Bct⁺/TH⁺
8/8
D
LED
Kglu
NE
NPY
No response
Outward
1/39
Single pulses
-50mV
38/39
Train stimulation
5Hz
31/31
20Hz
32/32
50Hz
30/30
E
LED
CsCl
NPY
NE
No response
Single pulse
-60mV
20/20
+40mV
12/12
F
sIPSCs frequency (Hz)
6
4
2
0
sIPSCs amplitude (pA)
50
40
30
20
10
0

Figure S5

Figure S5. No GABAergic or glutamatergic synaptic connectivity between peri-LC_{NPY} and LC_{NE} neurons

A) Schematic of sagittal mouse brain, depicting the location of bilateral virus injections used to drive CoChR expression in *NPY-cre* mice. After a period of virus incubation (≥ 5 weeks) extensive CoChR innervation (GFP, yellow) was observed in the pericoerulean region (TH, cyan), in brain slices prepared for electrophysiological recordings.

B) Schematic representation of experimental design. In brain slices containing CoChR, we confirmed that peri-LC_{NPY} neurons expressing CoChR (GFP^+ cell) directly respond with photocurrents to respective optical stimulation (1 ms, single or 20 Hz pulse train of 20 pulses), validating efficiency of the optogenetic construct. Recording pipet is indicated. Example traces are shown. Scale bar: 50 pA, 50 ms (single) and 100 ms (20 Hz).

C) Putative LC_{NE} neurons were identified based on shape, position, and electrical properties. *Top*: in a representative subset of cells, we recorded spontaneous firing before and after bath application of the $\alpha 2$ adrenergic receptor agonist clonidine (1 µM). Clonidine abolished spontaneous firing in the sampled cells, a characteristic response of LC_{NE} neurons. Paired t-test, $t(5)=4.79$, $P=0.005$. N=4 mice, n=6 cells. Scale bar: 10 mV, 200 ms. *Bottom*: in a subset of recorded cells, we validated LC_{NE} identity after biocytin fillings and post-hoc TH immunolabeling. All LC_{NE} neurons were confirmed to be TH^+ (8/8).

D) In brain slices containing CoChR-expressing peri-LC_{NPY} neurons, we recorded LC_{NE} postsynaptic responses to 1 ms LED-mediated blue light pulses. Recordings were performed in voltage-clamp configuration, at -50 mV, using a KGlu-based internal solution, to allow for detection of both glutamatergic (inward) and GABAergic (outward) responses. No postsynaptic responses to peri-LC_{NPY} photostimulation were observed in the vast majority of LC_{NE} cells (38/39), indicating next-to-null ionotropic receptor-mediated connectivity in the two populations. One cell showed a nominal outward, presumably GABA-mediated, response. Example traces are shown; scale bar: 5 mV, 10 ms. Blue rectangle: start of LED stimulation. In addition, LC_{NE} synaptic responses to pulse trains of optical stimulation paradigms were recorded. No postsynaptic responses were detected in any of the frequencies sampled (5 Hz, 31/31 cells; 20 Hz, 32/32; 50 Hz, 30/30 cells, non-responsive). Single pulse: N=14, n=39; 5 Hz: N=13, n=31; 20 Hz: N=13, n=32; 50 Hz: N=12, n=39.

E) In brain slices containing CoChR-expressing peri-LC_{NPY} neurons, we recorded LC_{NE} opto-responses at -60 mV or +40 mV, using a CsCl-based internal solution, to increase sensitivity in detection of $GABA_AR$, AMPAR, or NMDA-mediated currents. No response to photostimulation in any of the recording conditions was observed. -60 mV, N=6, n=20; +40 mV, N=3, n=12.

F) Recordings of spontaneous inhibitory postsynaptic currents (sIPSCs) in LC_{NE} neurons using a CsCl-based internal solution. Clear occurrence of spontaneous synaptic events can be observed, showing that absence of GABA/Glu connectivity between peri-LC_{NPY} and LC_{NE} cells and is not due to a general absence of measurable synaptic connectivity in the preparation. Scale bar: 50 pA, 100 ms. N=9 mice, n=17 cells.

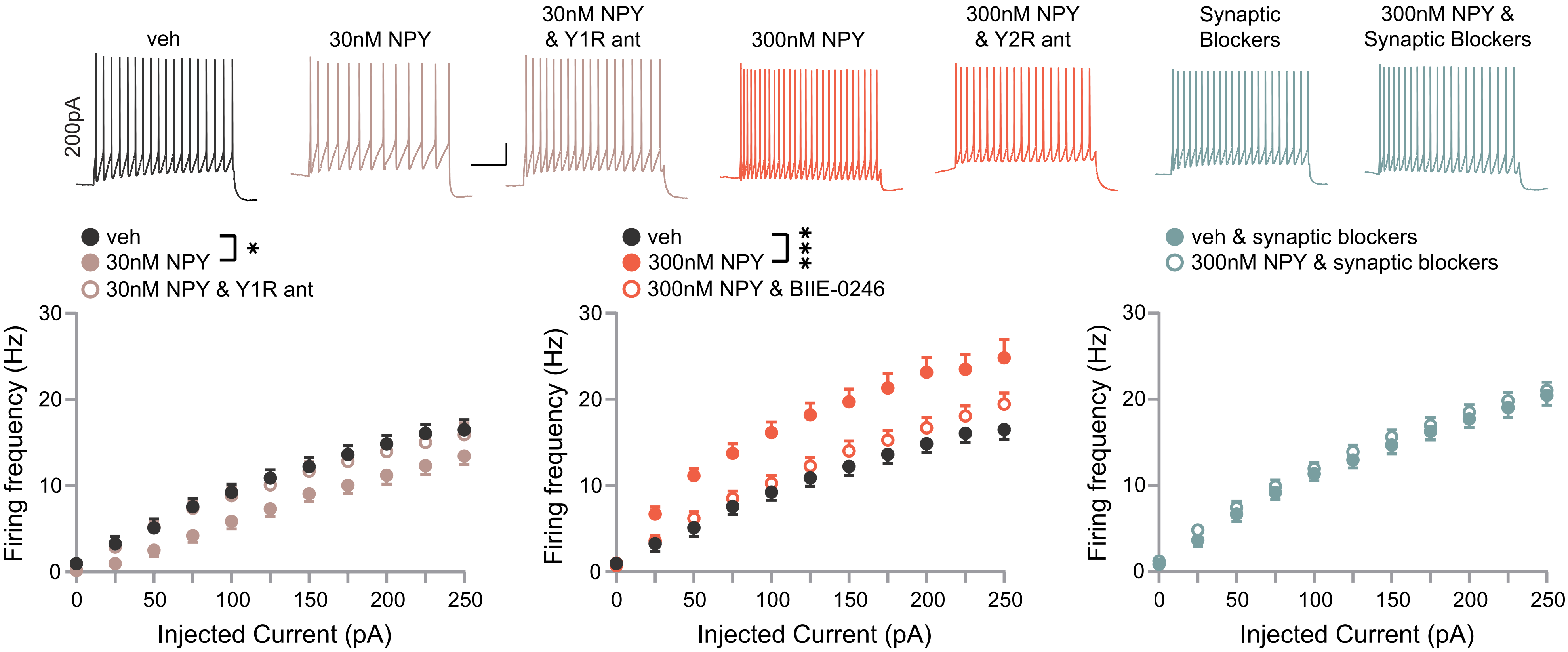
veh
30nM NPY
30nM NPY & Y1R ant
300nM NPY
300nM NPY & Y2R ant
Synaptic Blockers
300nM NPY & Synaptic Blockers
200pA
veh
30nM NPY
30nM NPY & Y1R ant
*
300nM NPY
300nM NPY & BIIE-0246
***
veh & synaptic blockers
300nM NPY & synaptic blockers
Firing frequency (Hz)
Injected Current (pA)
0
10
20
30
0
50
100
150
200
250

Figure S6

Figure S6. Effects of NPY bath application on LC_{NE} excitability

Brain slices from wild-type C57Bl/6 mice were prepared for electrophysiological recordings. Whole-cell patch clamp recordings from LC_{NE} neurons were conducted in current-clamp configuration, while different concentrations of NPY (or vehicle) were continuously perfused.

Left: In the presence of 30nM we detected fewer action potentials in response to increasing current injections as compared to vehicle, and this effect was blocked by pretreatment with the selective Y1R antagonist BIBO-3304 (1 μM, Firing frequency: 2-way RM ANOVA, main treatment effect, $F(2,45)=3.31$, $P=0.045$). Veh, N=4, n=15; 30 nM, N=3, n=15; 30 nM & BIBO-3304, N=3, n=18.

Middle: 300 nM NPY led to LC_{NE} hyper-excitability, which was abolished by pretreatment with the selective Y2R antagonist BIIE-0246 (1 μM, Firing frequency: 2-way RM ANOVA, main treatment effect, $F(2,39)=10.3$, $P=0.0003$). Veh, N=4, n=15; 300 nM, N=4, n=11; 300 nM & BIIE-0246, N=3, n=16.

Right: The effect of high NPY dose was dependent on presynaptic input to LC_{NE} cells, as pharmacological blockade of AMPARs and KainateRs (10 μM CNQX), NMDARs (50 μM D-AP5), $GABA_A$Rs (100 μM picrotoxin) and $GABA_B$Rs (10 μM CGP-54626) occluded NPY-driven increase in LC_{NE} firing frequency (2-way RM ANOVA, main treatment effect, $F(1,32)=0.48$, $P=0.494$). Veh & block, N=3, n=14; 300 nM NPY & block, N=3, n=20.

Representative traces of LC_{NE} firing in response to 200 pA current injection are depicted for all conditions. Scale bar 20 mV, 200 ms.

Data depicted as mean ± SEM. * $P<0.05$. *** $P<0.001$.

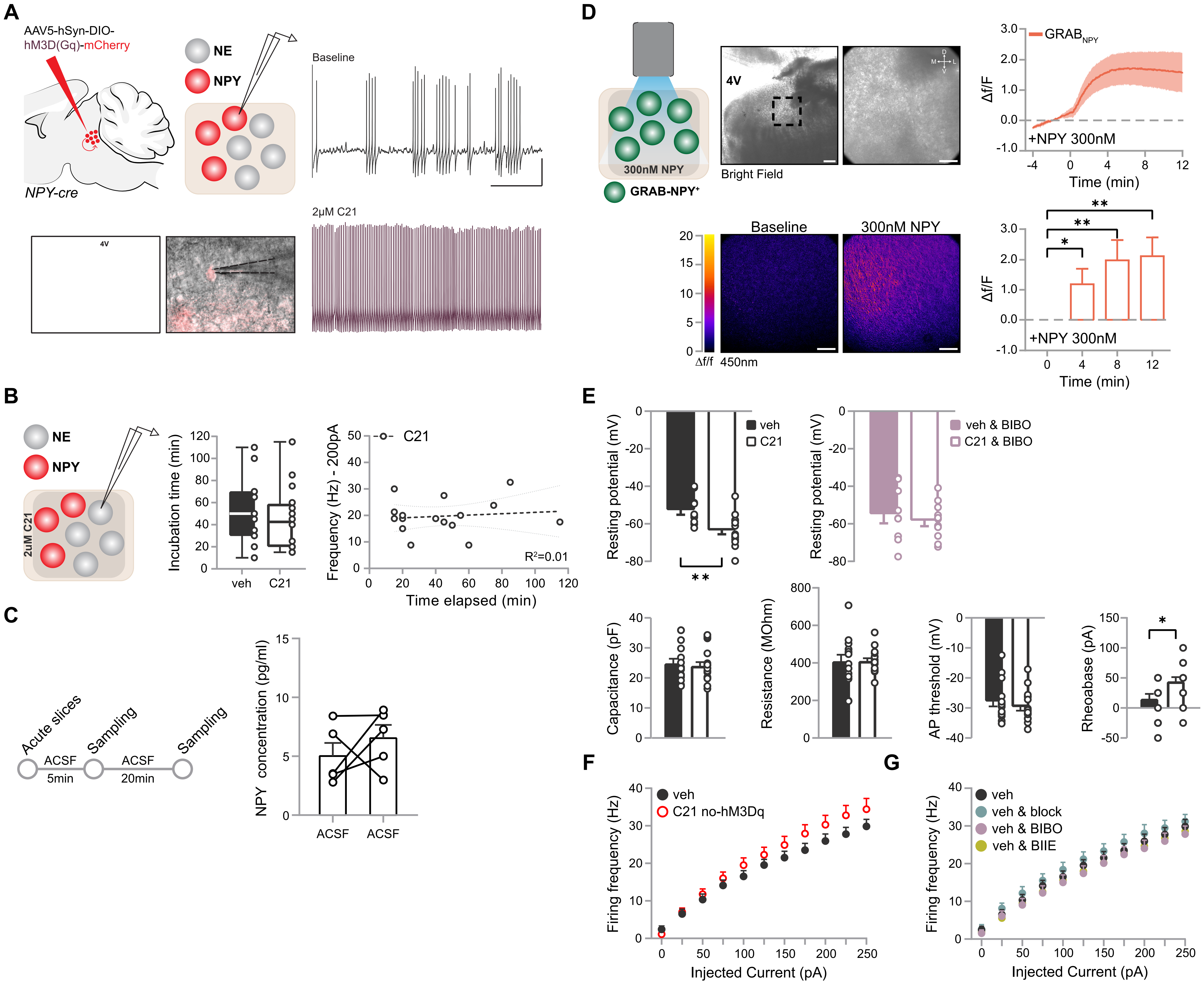
A
AAV5-hSyn-DIO-
hM3D(Gq)-mCherry
NPY-cre
NE
NPY
Baseline
2μM C21
4V
B
NE
NPY
2uM C21
Incubation time (min)
veh
C21
Frequency (Hz) - 200pA
C21
R²=0.01
Time elapsed (min)
C
Acute slices
Sampling
Sampling
ACSF
5min
ACSF
20min
NPY concentration (pg/ml)
ACSF
ACSF
D
4V
300nM NPY
GRAB-NPY⁺
Bright Field
GRAB_NPY
Δf/F
+NPY 300nM
Time (min)
Baseline
300nM NPY
Δf/f
450nm
E
Resting potential (mV)
veh
C21
veh & BIBO
C21 & BIBO
Capacitance (pF)
Resistance (MOhm)
AP threshold (mV)
Rheobase (pA)
F
Firing frequency (Hz)
veh
C21 no-hM3Dq
Injected Current (pA)
G
Firing frequency (Hz)
veh
veh & block
veh & BIBO
veh & BIIE
Injected Current (pA)

Figure S7

Figure S7. Effects of chemogenetic stimulation of peri-LC_{NPY} cells on local NPY release and intrinsic properties and excitability of LC_{NE} cells.

A) Schematic of bilateral virus injections for hM3D(Gq) in *NPY-cre* mice. Whole-cell patch clamp recordings of peri-LC_{NPY} cells were conducted in current-clamp mode. Representative images of location of the recorded cell (mCherry$^+$), the LC and the recording pipet are indicated (dashed lines). Representative traces of 2.5 s of spontaneous activity of a peri-LC_{NPY} cell before (top, grey) and after (bottom, magenta) bath application of the DREADD actuator C21 (2 µM) are included. Scale bar, 20 mV, 500 ms. C21 resulted in a substantial increase in the number of action potentials fired at rest.

B) Brain slices from *NPY-cre* mice, bilaterally expressing the excitatory hM3Dq DREADD, were prepared for electrophysiological recordings. LC_{NE} cell excitability was assessed minimum after 10 and up to a maximum of 115 min after C21 application. No differences in incubation time between vehicle and C21 were observed: Mann-Whitney U=1173, P=0.143, and both conditions had a median elapsed time (pharmacology application to cell recording) of 50 min. Regression analysis of LC_{NE} firing frequency at example current injection (200 pA) versus C21 incubation time. No correlation between time elapsed and firing frequency was observed: R^2=0.01, F(1,14)=0.19, P=0.669. N=4 mice, n=16 cells.

C) *Left:* Experimental design for NPY ELISA. *Right:* Concentration of biochemically-detected NPY in the absence of treatment (ACSF alone), controlling for time effects. ACSF, 5.02 pg/ml; ACSF, 6.54 pg/ml; unpaired t-test, t(8)=0.96, P=0.365. N=5 replicates / treatment, n=4 mice / replicate.

D) *Left:* Recordings of $GRAB_{NPY}$ signal changes upon bath application of NPY (300 nM) were performed in LC-containing brain slices. Representative images of [1] an acute brain slice containing the (peri) LC region (inset) in bright field. Scale bar 200 µm; [2] a higher magnification of this selected area in bright field. Scale bar 10 µm. [3] Same as [2], but with a color-coded heatmap of the $GRAB_{NPY}$ ΔF/F signal. For clarity the image is a subtracted average of 100 frames [4]. As [3], but during NPY application.

Right: Average ΔF/F over time after NPY bath application and quantification of changes in $GRAB_{NPY}$ ΔF/F from NPY superfusion (0 min) onward per 4 min bin. NPY-induced increase in $GRAB_{NPY}$ signal peaked 8 min after NPY. RM ANOVA, main time effect F(3,9)=10.99, P=0.002. Multiple comparisons: 0 to 4 min, P=0.045; 4 to 8 min, P=0.003; 8 to 12 min, P=0.002 versus baseline (-4 to 0 min).

E) Whole-cell patch clamp recordings from LC_{NE} neurons were conducted in current-clamp configuration. *Top*: Resting membrane potential of LC_{NE} neurons in the presence of C21 (2 µM): veh, -52.7 mV; C21, -63.5 mV; unpaired t-test, t(23)=3.30, P=0.003. Veh, N=4, n=15; C21, N=4, n=17. BIBO-3304 (1 µM) bath application reversed C21 effects: veh, -55.0 mV; C21, -60.6 mV; unpaired t-test, t(16)=1.74, P=0.100. Veh & BIBO-3304, N=3, n=13; C21 & BIBO-3304, N=4, n=13.

Bottom: No effects of peri-LC_{NPY} chemogenetic simulation on LC_{NE} cellular capacitance (veh, 24.8 pF *vs.* C21, 24.0 pF, Mann-Whitney U=117.5, P=0.716), membrane resistance (veh, 408.1 MOhm *vs.* C21, 407.9 MOhm, unpaired t-test, t(29)=0.04, P=0.965), or action potential threshold (veh, -27.8 mV *vs.* C21, -29.6 mV, Mann-Whitney U=108.5, P=0.484) were observed. In support of reduced LC_{NE} excitability (*cf.*, Fig. 2F), C21 increased the current necessary for LC_{NE} cells to exceed their action potential threshold and fire (Rheobase: veh,15.0 pA *vs.* C21, 44.1 pA, Mann-Whitney U=65, P=0.013). Veh, N=4 mice, n=15 cells; C21, N=4 mice, n=17 cells.

F) In brain slices of *NPY-cre* mice that did not express hM3Dq, we recorded LC_{NE} firing frequency in response to increasing currents in presence of C21 (2 µM). C21 alone did not alter LC_{NE} excitability (2-way RM ANOVA, main effect of treatment, F(1,23)=1.38, P=0.252), controlling for effects of unspecific binding of the DREADD actuator. Veh, N=4 mice, n=15 cells; C21, N=2 mice, n=10 cells.

G) Effects of receptor antagonism on LC_{NE} firing patterns. In vehicle-treated slices, pharmacological blockade of AMPAR-, NMDAR-, $GABA_AR$ and $GABA_BR$-mediated input or antagonism of Y1R and Y2R had no effect on LC_{NE} firing (2-way RM ANOVA, main effect of treatment, F(3,49)=0.71, P=0.550). Veh, N=4

mice, n=15 cells; Veh & block, N=2 mice, n=14 cells; Veh & BIBO-3304, N=3 mice, n=13 cells; Veh & BIIE-0246, N=2 mice, n=11 cells.

Data depicted as mean ± SEM. * $P < 0.05$.

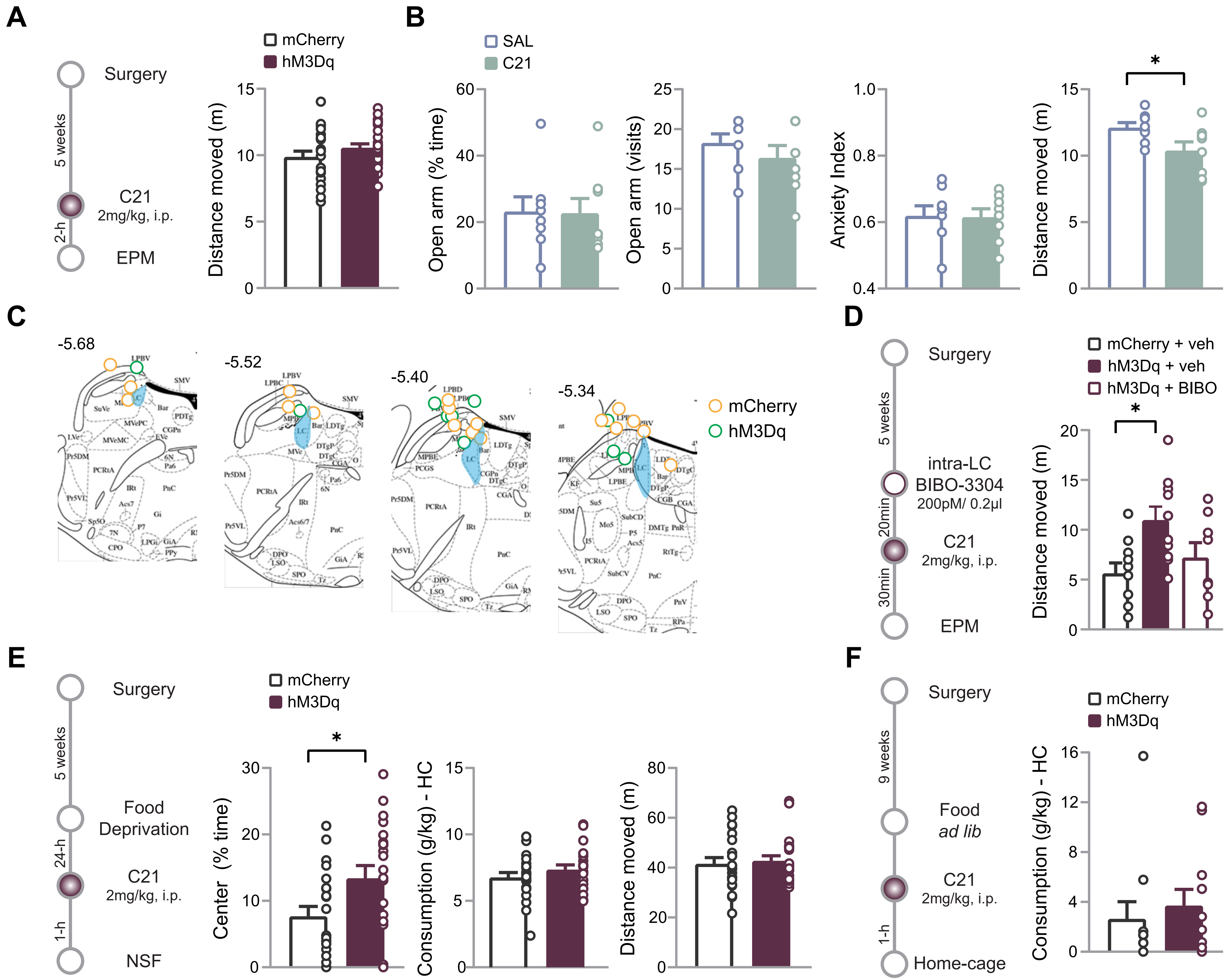
A
Surgery
5 weeks
C21
2mg/kg, i.p.
2-h
EPM
mCherry
hM3Dq
Distance moved (m)
B
SAL
C21
Open arm (% time)
Open arm (visits)
Anxiety Index
Distance moved (m)
*
C
-5.68
-5.52
-5.40
-5.34
mCherry
hM3Dq
D
Surgery
5 weeks
intra-LC
BIBO-3304
200pM/ 0.2µl
20min
C21
2mg/kg, i.p.
30min
EPM
mCherry + veh
hM3Dq + veh
hM3Dq + BIBO
Distance moved (m)
*
E
Surgery
5 weeks
Food
Deprivation
24-h
C21
2mg/kg, i.p.
1-h
NSF
mCherry
hM3Dq
Center (% time)
*
Consumption (g/kg) - HC
Distance moved (m)
F
Surgery
9 weeks
Food
ad lib
C21
2mg/kg, i.p.
1-h
Home-cage
mCherry
hM3Dq
Consumption (g/kg) - HC

Figure S8

Figure S8. Behavioral outcomes of peri-LC$_{NPY}$ chemogenetic activation and C21 administration

A) Experimental design for *in vivo* chemogenetic manipulations: After 5 weeks allowing for virus expression, hM3Dq and mCherry groups were systemically administered C21 (2 mg/kg, i.p.) and 2-h later subjected to the elevated plus maze (EPM) task. Chemogenetic activation of peri-LC$_{NPY}$ neurons had no consequences on general locomotor activity, as measured by total distance moved at the EPM task (mCherry, 9.9 m; hM3Dq, 10.6 m; unpaired t-test, t(40)=1.28, *P*=0.209). mCherry, N=21; hM3Dq, N=21.

B) Controlling for non-specific, off-target effects, administration of C21 (2 mg/kg, i.p.), did not alter anxiety-like phenotypes in the EPM as compared to vehicle (saline), in a separate cohort of wild-type C57BL/6 mice that did not express the hM3Dq-carrying viral construct: time spent in open arms (% of total exploration time): SAL, 23.2 %; C21, 22.7 %; unpaired t-test, t(14)=0.08, *P*=0.937); open arms entries: SAL, 18.2; C21, 16.4; unpaired t-test, t(14)=0.96, *P*=0.353); anxiety index: SAL, 0.62; C21, 0.62; unpaired t-test, t(14)= 0.09, *P*=0.926). We observed a slight decrease in maze exploration in mice administered C21 (Distance moved: SAL, 12.1 m; C21, 10.4 m; unpaired t-test t(14)=2.23, *P*=0.042). SAL, N=8; C21, N=8.

C) Animals were implanted with bilateral cannulas targeting the peri-LC for local pharmaca administration. Circles (mCherry, green; hM3Dq, yellow) indicate the position of the cannula tip for all animals that participated in the experiment (*cf.,* Fig. 3C, D), overlayed to the Paxinos & Franklin (2001) mouse brain atlas. The LC (blue) is indicated in the entire anterior-posterior axis.

D) In animals that received intra-LC micro-infusions via cannula, a significant main effect of group was observed in distance moved (1-way ANOVA, F(2,25)=4.71, *P*=0.018). C21 (2 mg/kg) altered locomotion in hM3Dq & veh group, which displayed increased distance moved (11 m) *vs.* mCherry & veh controls (5.6 m, *P*=0.016). No other statistically significant group differences were detected (mCherry & veh *vs.* hM3Dq & BIBO, 7.2 m, *P*=0.683; hM3Dq & veh *vs.* hM3Dq & BIBO, *P*=0.136). The observed discrepancy between A) and D) could be due to differences in experimental design, and in particular the time interval between C21 administration and EPM (2-h *vs.* 30-min, respectively). mCherry & veh, N=10; hM3Dq & veh, N=10; hM3Dq & BIBO-3304, N=8.

E) After 5 weeks allowing for hM3Dq expression, mice were food deprived (24-h) and systemically administered the DREADD agonist compound 21 (C21, 2 mg/kg, i.p.). One hour after after C21 injection, mice we subjected to the novelty suppressed feeding (NSF) task. hM3Dq group spent more time at the center of the NSF arena, containing a familiar food source, *vs.* mCherry controls (Duration Center: mCherry, 7.7 s; hM3Dq, 13.4 s; Mann-Whitney U=137, *P*=0.035). Chemogenetic activation of peri-LC$_{NPY}$ neurons did not affect general consummatory behavior, as no group differences in home-cage (HC) food intake were seen (mCherry, 6.76 g/kg; hM3Dq, 7.35 g/kg; unpaired t-test, t(40)=1.14, *P*=0.261). No unspecific effects of C21 in general locomotion were seen (Distance moved: mCherry 41.5 m, hM3Dq, 42.7 m; Mann-Whitney U=202, *P*=0.654). mCherry, N=21; hM3Dq, N=21.

F) After nine weeks allowing for virus expression, hM3Dq and mCherry groups were systemically administered C21 (2mg/kg, i.p.) and returned to their home-cage, where food (standard laboratory chow) was available *ad libitum*. One hour following C21 injection, food was weighted, and its consumption in the home-cage was monitored for a total of 3-h. Under conditions of satiety, C21 did not affect consummatory behavior in the hM3Dq group *vs*. mCherry controls (mCherry 2.63 g/kg, hM3Dq, 3.70 g/kg; Mann-Whitney U=48, *P*=0.425). mCherry, N=11; hM3Dq, N=11.

Data depicted as mean ± SEM. * *P* <0.05.

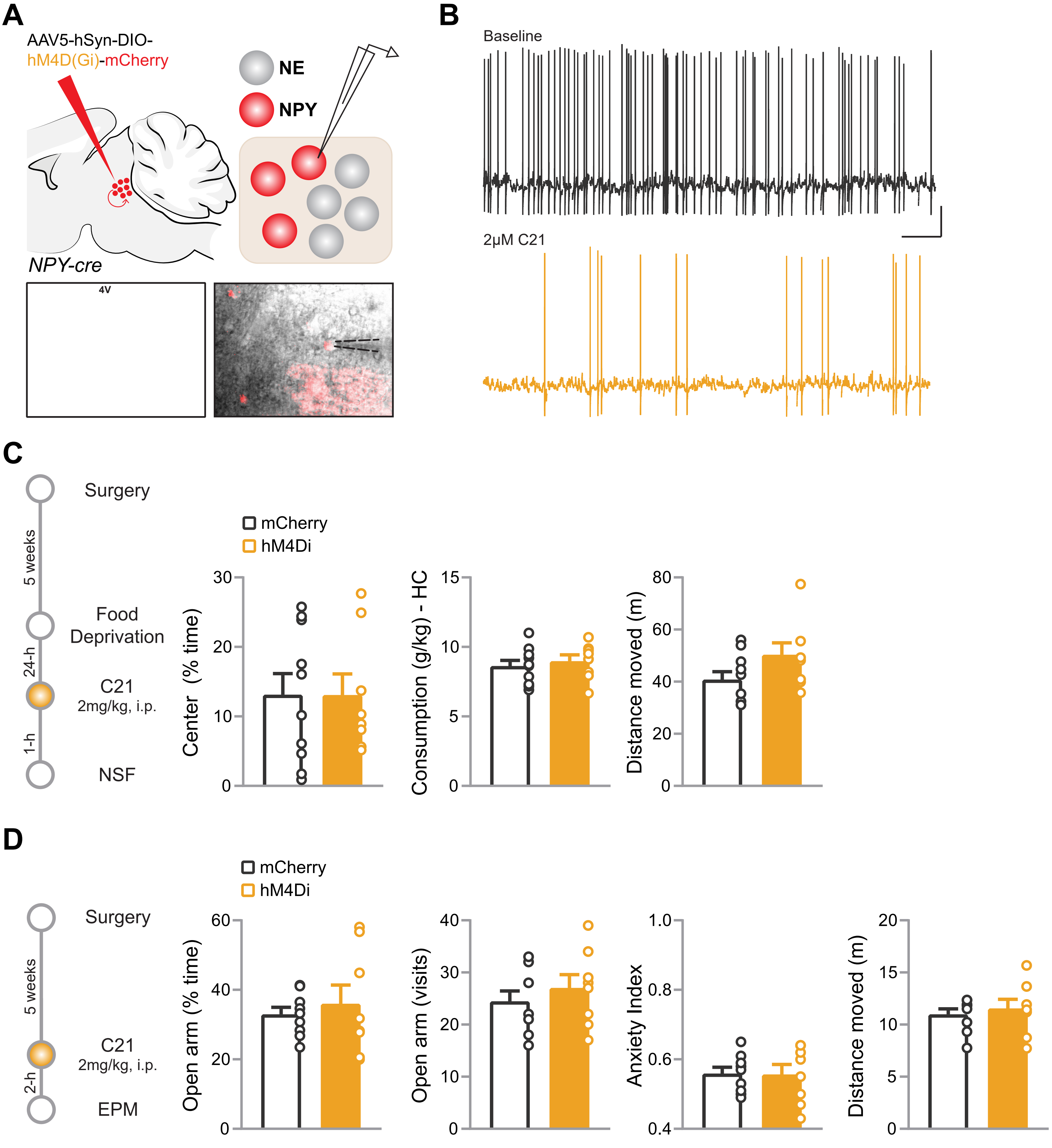
A
AAV5-hSyn-DIO-
hM4D(Gi)-mCherry
NE
NPY
NPY-cre
4V
B
Baseline
2μM C21
C
Surgery
5 weeks
Food
Deprivation
24-h
C21
2mg/kg, i.p.
1-h
NSF
mCherry
hM4Di
Center (% time)
Consumption (g/kg) - HC
Distance moved (m)
D
Surgery
5 weeks
C21
2mg/kg, i.p.
2-h
EPM
mCherry
hM4Di
Open arm (% time)
Open arm (visits)
Anxiety Index
Distance moved (m)

Figure S9

Figure S9. Behavioral outcomes of peri-LC_{NPY} chemogenetic inhibition

A) Schematic of sagittal mouse brain depicting the location of bilateral virus injections used to drive hM4D(Gi) in *NPY-cre* mice. After a period allowing for virus expression (≥5 weeks), brain slices containing the LC were prepared and whole-cell patch clamp recordings of peri-LC_{NPY} cells were conducted in current-clamp mode. Representative images of location of the recorded cell ($mCherry^{+}$) are included; LC and recording pipet are indicated (dashed lines).

B) Representative traces of 30 s of spontaneous activity of a peri-LC_{NPY} cell before (top, grey) and after (bottom, orange) C21 (2 µM) bath application (Scale bar, 20 mV, 2.5 s). C21 resulted in a substantial decrease in the number of action potentials fired at rest.

C) Experimental design for *in vivo* chemogenetic manipulations: after 5 weeks allowing for virus expression, mice were food deprived (24-h) and systemically administered the DREADD agonist compound 21 (C21, 2 mg/kg, i.p.). One hour after C21 injection, mice we subjected to the novelty suppressed feeding (NSF) task. In hM4Di mice, C21 did not alter time spent at the center of the NSF arena as compared to mCherry controls (Duration Center: mCherry, 13.1 s; hM4Di, 13.0 s; unpaired t-test t(16)=0.01, *P*=0.988). C21 effects on consummatory behavior were specific for the anxiogenic context (*cf.*, Fig. 3H), as chemogenetic inhibition of peri-LC_{NPY} neurons did not affect general consummatory behavior (Home-cage food intake: mCherry, 8.62 g/kg; hM4Di, 8.97 g/kg; unpaired t-test, t(16)=0.57, *P*=0.575). Finally, C21 did not significantly affect general locomotion in hM4Di mice (Distance moved: mCherry, 40.8 m; hM4Di, 50.3 m; unpaired t-test, t(16)=1.78, *P*=0.094). mCherry, N=10; hM4Di, N=8.

D) hM4Di and mCherry groups were systemically administered C21 (2 mg/kg, i.p.) and 2-h later subjected to the elevated plus maze (EPM) task. In contrary to NSF, we observed no anxiogenic effects of peri-LC_{NPY} silencing on EPM performance: time spent in open arms (% of total exploration time): mCherry, 32.9 %; hM4Di, 36 %; unpaired t-test, t(15)=0.56, *P*=0.585); open arms entries: mCherry, 24.4; hM4Di, 27; unpaired t-test, t(15)=0.79, *P*=0.442); anxiety index: mCherry, 0.56; hM4Di, 0.56; unpaired t-test, t(15)=0.08, *P*=0.938). C21 did not affect general locomotor activity in EPM (Distance moved: mCherry, 11 m; hM4Di, 11.5 m; unpaired t-test, t(15)=0.55, *P*=0.590). mCherry, N=9; hM4Di, N=8.

Data depicted as mean ± SEM.

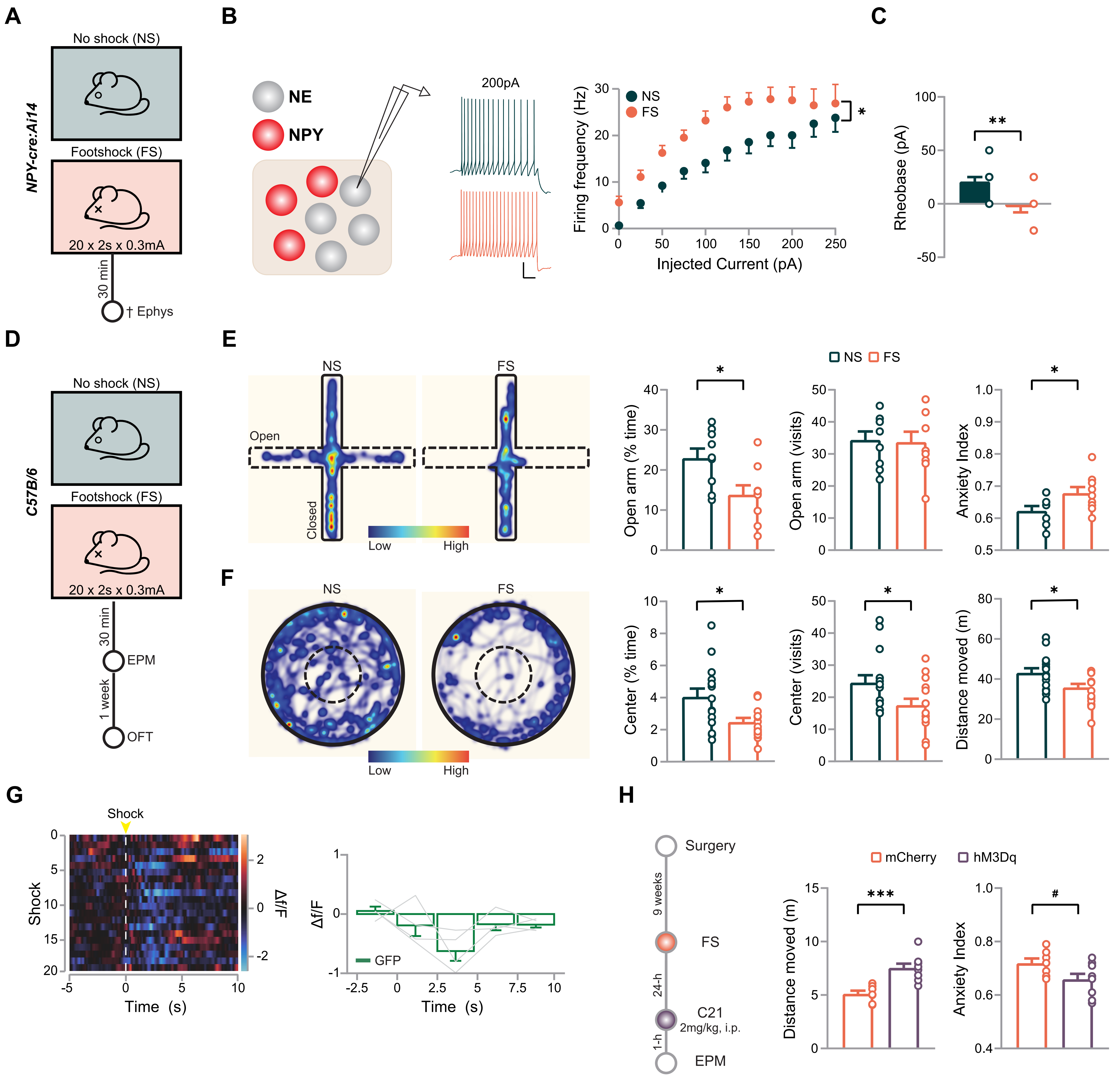

A
B
C
D
E
F
G
H
NPY-cre:Ai14
No shock (NS)
Footshock (FS)
20 x 2s x 0.3mA
30 min
† Ephys
NE
NPY
200pA
Firing frequency (Hz)
Injected Current (pA)
NS
FS
Rheobase (pA)
C57B/6
EPM
1 week
OFT
Open
Closed
Low
High
Open arm (% time)
Open arm (visits)
Anxiety Index
Center (% time)
Center (visits)
Distance moved (m)
Shock
Time (s)
Δf/F
GFP
Surgery
9 weeks
24-h
C21
2mg/kg, i.p.
1-h
mCherry
hM3Dq

Figure S10

Figure S10. Foot-shock stress recruits LC_{NE} neurons and triggers lasting anxiety-like phenotypes

A) Experimental design: control (NS) or foot-shock (FS)-subjected (20 shocks x 2 s x 0.3 mA) *NPY-cre:Ai14* mice were sacrificed 30 min following stress or novel context exposure for electrophysiological recordings.

B) Whole-cell patch clamp recordings from LC_{NE} neurons. Stress exposure increased LC_{NE} firing frequency, as seen in the number of action potentials fired in response to increasing current injections (2-way RM ANOVA, main stress effect: $F(1,26)=9.27$, $P=0.005$). Representative example traces of action potentials fired in response to a 200 pA current injection in NS and FS mice are depicted. Scale bar: 20 mV, 100 ms. NS, N=5, n=12; FS, N=6, n=16.

C) Stress reduced rheobase in the FS group (NS, 25 pA, FS, 0 pA, Mann-Whitney U=41, P=0.006), further corroborating increased LC_{NE} neuronal excitability. NS, N=5, n=12; FS, N=6, n=16.

D) Experimental design: acute (30 min, elevated plus maze -EPM) and long-lasting (1-week, open field test -OFT) effects of stress were assessed in wild-type C57BL/6 mice.

E) Stress exposure acutely increased anxiety-like phenotypes in the EPM. Stressed mice spent less time in the open arms of the maze when compared to controls (NS, 23 % *vs.* FS, 13.8 % of total EPM time; unpaired t-test, $t(16)=2.71$, $P=0.016$). We observed no stress effects on the frequency of visits to the open arms (NS, 34.3 *vs.* FS, 33.7; unpaired t-test, $t(16)=0.16$, $P=0.878$). Nevertheless, stress increased anxiety index (NS, 0.62 *vs.* FS, 0.68; unpaired t-test, $t(16)=2.26$, $P=0.038$). Representative spatial location heatmaps show time spent exploring the EPM. NS, N=9; FS, N=9.

F) Stress exposure led to long-lasting anxiety-like phenotypes in the OFT. The FS group spent less time in the center of the open field arena (NS, 4.0 % *vs.* FS, 2.4 % of total OFT time; unpaired t-test, $t(28)=2.727$, $P=0.011$) and displayed reduced frequency of visits to the center (NS, 24.5 *vs.* FS, 17.5; unpaired t-test, $t(28)=2.286$, $P=0.030$). Stress had a lingering effect on explorative behavior in the OFT arena (Distance moved: NS, 43 m *vs.* FS, 35.7 m; unpaired t-test, $t(28)=2.486$, $P=0.019$). Representative spatial location heatmaps show time spent exploring the open OF arena. NS, N=15, FS, N=15.

G) *Left*: Representative heatmap of GFP (control construct) $\Delta F/F$ response time-locked to foot-shock (0s). *Right*: Quantification of changes in $\Delta F/F$ from shock (0s) onward per 2.5 s bin: RM ANOVA, main time effect $F(3,12)=1.47$, $P=0.071$. N=4.

H) Experimental design: after 9 weeks allowing for virus expression, mice were subjected to foot-shock stress. One day after stress exposure, mice were systemically administered C21 (2mg/kg, i.p.). One hour after C21 injection, mice participated in an EPM test. In hM3Dq mice C21 increased general explorative behavior, as compared to mCherry controls (mCherry, 5.1 m; hM3Dq, 7.5 m; unpaired t-test, $t(14)=4.65$, $P=0.000$). A trend for reduced anxiety index was observed: mCherry 0.72; hM3Dq, 0.66; unpaired t-test, $t(14)=2.12$, $P=0.052$. mCherry, N=7; hM3Dq, N=9.

Data depicted as mean ± SEM. * $P<0.05$, ** $P<0.01$, *** $P<0.001$.

**Brain Region**	***Cell Bodies***	**Brain Region**	***Cell Bodies***
mPFC	-	LPA	++
Acb	-	Arc	-
BLA	-	DMH	+
CeA	+	LH	++
BNST	+/-	PH	++
HPC	-	SN	+
LS	-	LC	**++**
Hb	-	LDTg	-
mTh	++	RtTg	+
LD	+	PBN	+
PV	+/-	PAG	++
MPA	++	DR	++

Table S1. Retrograde labelling in *NPY-cre* mice

Expression of mCherry$^+$ cell bodies was examined throughout the brain following bilateral injections of HSV-hEf1a-LS1L-mCherry virus targeting the LC of *NPY-cre* mice (N=5). (Pre)frontal cortical areas were devoid of retrograde labelling. Besides the pericoerulean region (*c.f.*, Fig. 1, Fig. S4), strong expression was observed in several hypothalamic nuclei as well as in the pons.

mPFC, medial prefrontal cortex; Acb, nucleus accumbens; BLA, basolateral amydgala; CeA, central amygdala; BNST, bed nucleus of the stria terminalis; HPC, hippocampus; LS, lateral septum; Hb, habenula; LD, laterodorsal thalamic nucleus; PV, paraventricular thalamic nucleus; MPA, medial preoptic area, LPA, lateral preoptic area; Arc, arcuate hypothalamic nucleus; DM, dorsal medial hypothalamus; LH, lateral hypothalamic area; PH, posterior hypothalamic area; SN, substantia nigra; LDTg, laterodorsal tegmental nucleus; LC: locus coeruleus; RtTg, reticulotegmental nucleus of the pons; PBN, parabrachial nucleus; PAG, periaqueductal gray; dorsal raphe, DR. Plus (+) and minus (-) symbols represent the degree of mCherry expression observed. Terminology for presence of retrogradely labeled NPY+ cell bodies: - no cell bodies, +/- minor presence, + moderate presence, ++ extensive presence.

Readout	*Cell type*	*Group average*	*Statistics*	*P-value*
**Capacitance (pF)**	Peri-LC_{NPY}	NS: 9.6, FS: 10.6	t(84)=1.44	0.153
	LC_{NE}	NS: 32.9, FS: 22.9	t(25)=3.47	**0.002
**Membrane resistance (mOhm)**	Peri-LC_{NPY}	NS: 365.9, FS: 417.2	U=682	0.144
	LC_{NE}	NS: 404.6, FS: 328.9	t(24)=1.36	0.187
**Rheobase (pA)**	Peri-LC_{NPY}	NS: 25, FS: 25	U=906.5	0.898
	LC_{NE}	NS: 25, FS: 0pA	U=41	**0.006
**Action potential Threshold (mV)**	Peri-LC_{NPY}	NS: -34.7, FS: -35.2	U=846	0.525
	LC_{NE}	NS: -27.9; FS: -30.8	t(26)=1.28	0.210
**Resting membrane potential (mV)**	Peri-LC_{NPY}	NS: -67.4, FS: -64.9	t(84)=0.86	0.393
	LC_{NE}	NS: -50.4, FS: -54.3	t(13)=0.75	0.465

Table S2. Stress effects on LC_{NE} and peri-LC_{NPY} cells electrophysiological profile

Other than changes in firing profile (*cf*. Fig. 4), we detected no effects of exposure to foot-shocks on peri-LC_{NPY} neurons intrinsic electrophysiological properties. Stress decreased cellular capacitance and rheobase in LC_{NE} neurons. Peri-LC_{NPY}: NS, N=10, n=40; FS, N=13, n=46; LC_{NE}: NS, N=5, n=12, FS, N=6, n=16.

Data S1. Source data underlying the findings presented in Main and Supplemental Figures & Tables.
